# NECAP 1 Regulates AP-2 Interactions to Control Vesicle Size, Number, and Cargo During Clathrin-Mediated Endocytosis

**DOI:** 10.1371/journal.pbio.1001670

**Published:** 2013-10-01

**Authors:** Brigitte Ritter, Sebastian Murphy, Hatem Dokainish, Martine Girard, Manasa V. Gudheti, Guennadi Kozlov, Marilene Halin, Jacynthe Philie, Erik M. Jorgensen, Kalle Gehring, Peter S. McPherson

**Affiliations:** 1 Department of Neurology and Neurosurgery, Montreal Neurological Institute, McGill University, Montreal, Quebec, Canada; 2 Department of Biochemistry, Boston University School of Medicine, Boston, Massachusetts, United States of America; 3 Department of Biochemistry, Groupe de Recherche Axé sur la Structure des Protéines, McGill University, Montreal, Quebec, Canada; 4 Howard Hughes Medical Institute, Department of Biology, University of Utah, Salt Lake City, Utah, United States of America; 5 Vutara, Inc., Salt Lake City, Utah, United States of America; University of Texas Southwestern Medical Center, United States of America

## Abstract

AP-2 is the core-organizing element in clathrin-mediated endocytosis. During the formation of clathrin-coated vesicles, clathrin and endocytic accessory proteins interact with AP-2 in a temporally and spatially controlled manner, yet it remains elusive as to how these interactions are regulated. Here, we demonstrate that the endocytic protein NECAP 1, which binds to the α-ear of AP-2 through a C-terminal WxxF motif, uses an N-terminal PH-like domain to compete with clathrin for access to the AP-2 β2-linker, revealing a means to allow AP-2–mediated coordination of accessory protein recruitment and clathrin polymerization at sites of vesicle formation. Knockdown and functional rescue studies demonstrate that through these interactions, NECAP 1 and AP-2 cooperate to increase the probability of clathrin-coated vesicle formation and to control the number, size, and cargo content of the vesicles. Together, our data demonstrate that NECAP 1 modulates the AP-2 interactome and reveal a new layer of organizational control within the endocytic machinery.

## Abstract 2

The endocytic protein NECAP 1 cooperates with the endocytic adapter protein AP-2 to modulate interactions with accessory proteins and clathrin and to control the size, number, and cargo content of clathrin-coated vesicles.

## Author Summary

Clathrin-mediated endocytosis is the major entry portal for cargo molecules such as nutrient and signaling receptors in eukaryotic cells. Generation of clathrin-coated vesicles involves a complex protein machinery that both deforms the membrane to generate a vesicle and selects appropriate cargo. The endocytic machinery is formed around the core endocytic adapter protein AP-2, which recruits clathrin and numerous accessory proteins to the site of vesicle formation in a temporally and spatially controlled manner. Yet it remains elusive as to how these interactions are regulated to ensure efficient vesicle formation. Here we identify the endocytic protein NECAP 1 as a modulator of AP-2 interactions. We show that NECAP 1 and AP-2 form two functionally distinct complexes. In the first, NECAP 1 binds to two sites on AP-2 in such a manner as to limit accessory protein binding to AP-2. Recruitment of clathrin to vesicle formation sites displaces NECAP 1 from one of these sites, leading to the formation of a second complex in which NECAP 1 and AP-2 cooperate for efficient accessory protein recruitment. Through these interactions, NECAP 1 fine-tunes AP-2 function and the two proteins cooperate to increase the probability that a vesicle will form and to determine the size and cargo content of the resulting vesicle.

## Introduction

Clathrin-mediated endocytosis is the major vesicular entry route in mammalian cells. The formation of endocytic clathrin-coated vesicles (CCVs) depends on a complex protein machinery that deforms the planar plasma membrane into small, cargo-laden vesicles that are released into the cytosol [1],[2]. The endocytic machinery is organized around AP-2, a heterotetrameric protein complex in which the N-terminal regions of its two large subunits, α and β2, together with the two smaller subunits, σ2 and μ2, form a large globular domain referred to as the trunk [3]. The C-terminal region of each large subunit forms a small, bi-lobed globular domain termed the ear, and the two ears connect to the trunk through flexible linkers. Each of these regions mediates a specific function of the complex, allowing AP-2 to control the recruitment of a myriad of endocytic accessory proteins, cargo, and clathrin to PI(4,5)P_2_-rich sites of CCV formation at the plasma membrane [1],[2].

At the protein level, the endocytic machinery is built on the basic principle that short peptide motifs in unstructured or loosely structured regions of one protein mediate low-affinity interactions with a globular domain in a second protein [4]–[6]. In isolation, each interaction is of very low affinity and easily reversible; however, each protein has the potential to simultaneously interact with a number of binding partners to create an interaction network that stabilizes the protein coat around the forming vesicle.

Perhaps the least understood step of CCV formation is vesicle initiation, with two main models proposed to explain how new clathrin-coated pits (CCPs) are nucleated. In one, the FCHo complex, formed by FCHo1/2, Eps15, and intersectin, is recruited to PI(4,5)P_2_-rich sites at the plasma membrane, marking these sites for subsequent recruitment of clathrin/AP-2 [7],[8]. In the other, pits initiate by arrival of clathrin/AP-2 to PI(4,5)P_2_-rich sites with the FCHo complex recruited subsequently [9]. It is likely that in either scenario, clathrin/AP-2 complexes will need to be linked to the FCHo complex; however, the mechanisms that allow for efficient interconnection of the two complexes remain elusive.

Each endocytic accessory protein contributes to one or more specific aspects of vesicle formation such as membrane deformation, cargo recruitment, vesicle size control, and vesicle scission; thus, each needs to gain access to vesicle formation sites in the correct temporal order [2]. Many proteins target these sites through interactions with the globular ear domains of AP-2. During the course of vesicle formation, the β2-ear transitions from accessory protein binding to recruiting clathrin in conjunction with the β2-linker [10]–[12], whereas the α-ear serves as the main interface for accessory protein recruitment throughout the process [13]–[15]. All α-ear binding partners use one or more of three distinct peptide motifs to bind the ear; DPF/W and FxDxF motifs target the platform subdomain of the α-ear, while WxxF motifs bind the sandwich subdomain [6],[16]. Yet the mechanisms that modulate α-ear interactions allowing for appropriate recruitment of accessory proteins remain unknown.

In many cases, cargo selection and concentration in forming vesicles also depend on AP-2 [1]. Some cargo, such as the transferrin receptor, interact directly with the trunk region of AP-2. Other cargo, such as the low-density lipoprotein receptor, interact with alternate cargo adaptors, a heterogeneous subclass of accessory proteins that in turn interact with AP-2 and/or clathrin to bridge their cargo to the endocytic machinery [1],[17]. Therefore, mechanisms that control accessory protein recruitment to forming vesicles also provide a means to modulate vesicle cargo.

We identified NECAP 1 and 2 as CCV-enriched proteins using subcellular proteomics [18],[19]. The two proteins are only 62% identical at the amino acid level, and they function in distinct membrane trafficking processes. Knockdown (KD) of NECAP 2, a ubiquitously expressed isoform, does not appear to influence clathrin-mediated endocytosis but instead inhibits endosomal sorting (our preliminary data). NECAP 1, which functions in clathrin-mediated endocytosis [19], is primarily expressed in neurons but is also readily detected in cultured cell lines (Figure S1), which offer an easy-to-manipulate system to probe for its mechanistic role in this process. Through a WxxF motif at its C-terminus, NECAP 1 binds with high affinity to the α-ear sandwich subdomain of AP-2 [20]. The NECAP 1 N-terminus is well conserved with NECAP 1 orthologs in other species and encodes a globular PH fold, termed PHear [21]. PHear binds to accessory proteins harboring FxDxF motifs, similar to the platform subdomain of the AP-2 α-ear [21]. NECAP 1 binding to the α-ear sandwich site positions PHear in proximity of the α-ear platform, suggesting that NECAP 1/AP-2 complexes act synergistically to control the recruitment of FxDxF-containing accessory proteins to forming vesicles. In the current study we set out to examine the mechanistic role of NECAP 1 in endocytosis and we now report that NECAP 1 works cooperatively with AP-2 to recruit endocytic accessory proteins that control the number, size, and cargo content of CCVs.

## Results

### NECAP 1 Has Multiple Binding Sites for AP-2

**Figure 1:**
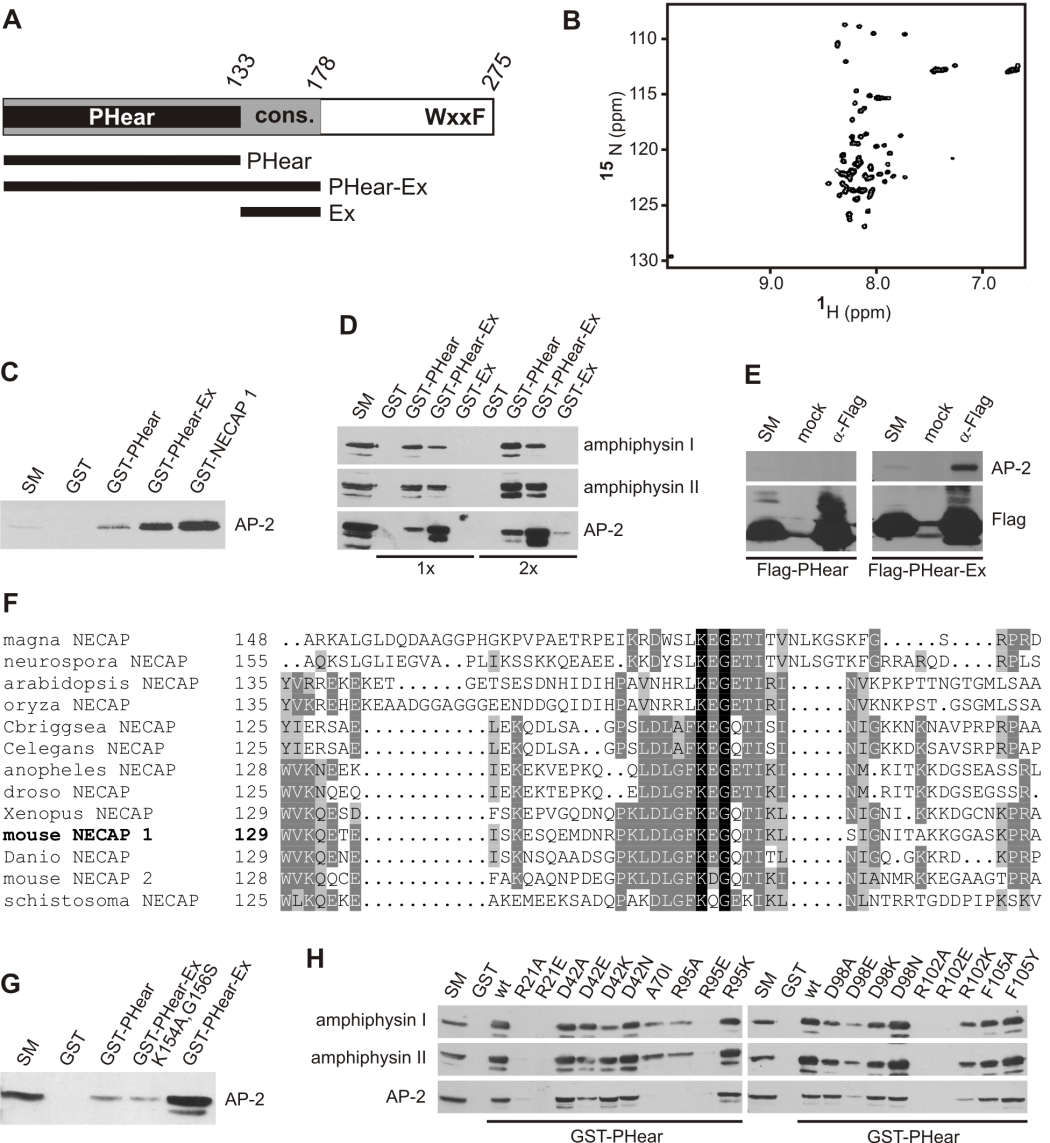
The conserved NECAP 1 N-terminus contributes to AP-2 binding. (A) Schematic representation of NECAP 1 and deletion variants. The conserved region is in grey, which includes the PHear domain in black, and the C-terminal WxxF-Ac motif is indicated. The numbers represent amino acid positions. (B) ^1^H-^15^N HSQC of NECAP 1 Ex. (C) Affinity-selection of endogenous AP-2 from COS-7 cells using purified GST or GST fused to PHear, PHear–Ex, or NECAP 1. (D) Affinity-selection of amphiphysin I/II and AP-2 from brain lysate using purified GST or GST fused to PHear, PHear–Ex, or Ex. Relative amounts of bait proteins used are indicated (1×, 2×). (E) Flag-tagged PHear and PHear–Ex were immunoprecipitated from transfected HEK-293–T cells using Flag antibody, and co-immunoprecipitation of endogenous AP-2 was assessed by Western blot. (F) Affinity-selection of AP-2 from brain lysate using purified GST or GST fused to PHear, PHear–Ex, or PHear–Ex K154A, G156S. (G) Sequence alignment of NECAP Ex (mouse residues 129–178) from different species as indicated: magna, *Magnaporthe oryzae*; neurospora, *Neurospora crassa*; arabidopsis, *Arabidopsis thaliana*; oryza, *Oryza sativa*; Cbriggsea, *Caenorhabditis briggsae*; Celegans, *Caenorhabditis elegans*; anopheles, *Anopheles gambiae*; droso, *Drosophila melanogaster*; Xenopus, *Xenopus laevis*; Danio, *Danio rerio*; schistosoma, *Schistosoma japonicum*. The numbers indicate amino acid positions within the respective protein. (H) Affinity-selection of amphiphysin I/II and AP-2 from brain lysate using purified GST or GST fused to wild-type PHear (wt) or PHear mutants as indicated. In (C–F) and (H), starting material (SM) represents 10% of the protein amount used in each binding.

The N-terminal region of NECAP 1 (aa 1–178) is highly conserved in NECAP 1 orthologs in other species, and residues 1–133 encode a PH-like domain [21]. We termed the domain PHear, as it has a PH fold and interacts with FxDxF motifs similar to the AP-2 α-ear [21]. Since the conservation extends beyond the C-terminal border of PHear (aa 129–178, termed Ex, Figure 1A), we reasoned that Ex could also have an important functional role. Ex shows no structural organization by NMR, on its own (Figure 1B) or in tandem with PHear [21]. Interestingly, when the entire conserved region of NECAP 1 (aa 1–178, termed PHear–Ex, Figure 1A) was used in affinity-selection assays from rat brain lysate, mass spectrometry of the isolated proteins identified multiple subunits of AP-2 (unpublished data). As the C-terminal WxxF motif that mediates NECAP 1 binding to the α-ear is not located within PHear–Ex (Figure 1A), these data indicate that PHear and/or Ex provide a second mechanism for NECAP 1 to engage AP-2. Western blot analysis confirmed that endogenous AP-2 is affinity-selected by GST–PHear–Ex (Figure 1C). AP-2 binding is stronger with PHear–Ex than PHear alone (Figure 1C), suggesting the presence of two AP-2 binding sites in the conserved N-terminus of NECAP 1. This was confirmed using PHear and Ex in isolation, which both bind AP-2 (Figure 1D). The observation that AP-2 binding to PHear–Ex is greater than the combined binding of PHear and Ex in isolation (Figure 1D) indicates that the two binding sites cooperate in AP-2 interaction. This is underscored by the fact that Flag-tagged PHear–Ex co-immunoprecipitates endogenous AP-2 from 293-T cell lysates while Flag-tagged PHear alone does not (Figure 1E). Thus, NECAP 1 has three distinct AP-2 binding sites, the high affinity WxxF motif at the C-terminus, and two lower affinity AP-2 binding sites in the conserved N-terminus, one in PHear and one in Ex.

To better define the interaction of PHear and Ex with AP-2, we first mutated K154 and G156, the only two amino acids within Ex that are invariant throughout evolution (Figure 1F). This double point mutation reduced AP-2 binding of PHear–Ex to levels seen with PHear alone (Figure 1G), further supporting the presence of a site in Ex that interacts with AP-2. As for PHear, we tested an array of previously generated PHear variants that contain mutations disrupting interactions with FxDxF motifs [21]. With only a few exceptions, mutations that reduce or eliminate binding to FxDxF motif proteins such as amphiphysin I and II also interfere with AP-2 binding (Figure 1H). Therefore, PHear uses an overlapping interface for binding to FxDxF motif proteins and AP-2, indicating that during CCV formation, NECAP 1 may transition between different roles by changing PHear binding partners.

### The PHear domain competes with clathrin for access to the β2-linker in AP-2

**Figure 2:**
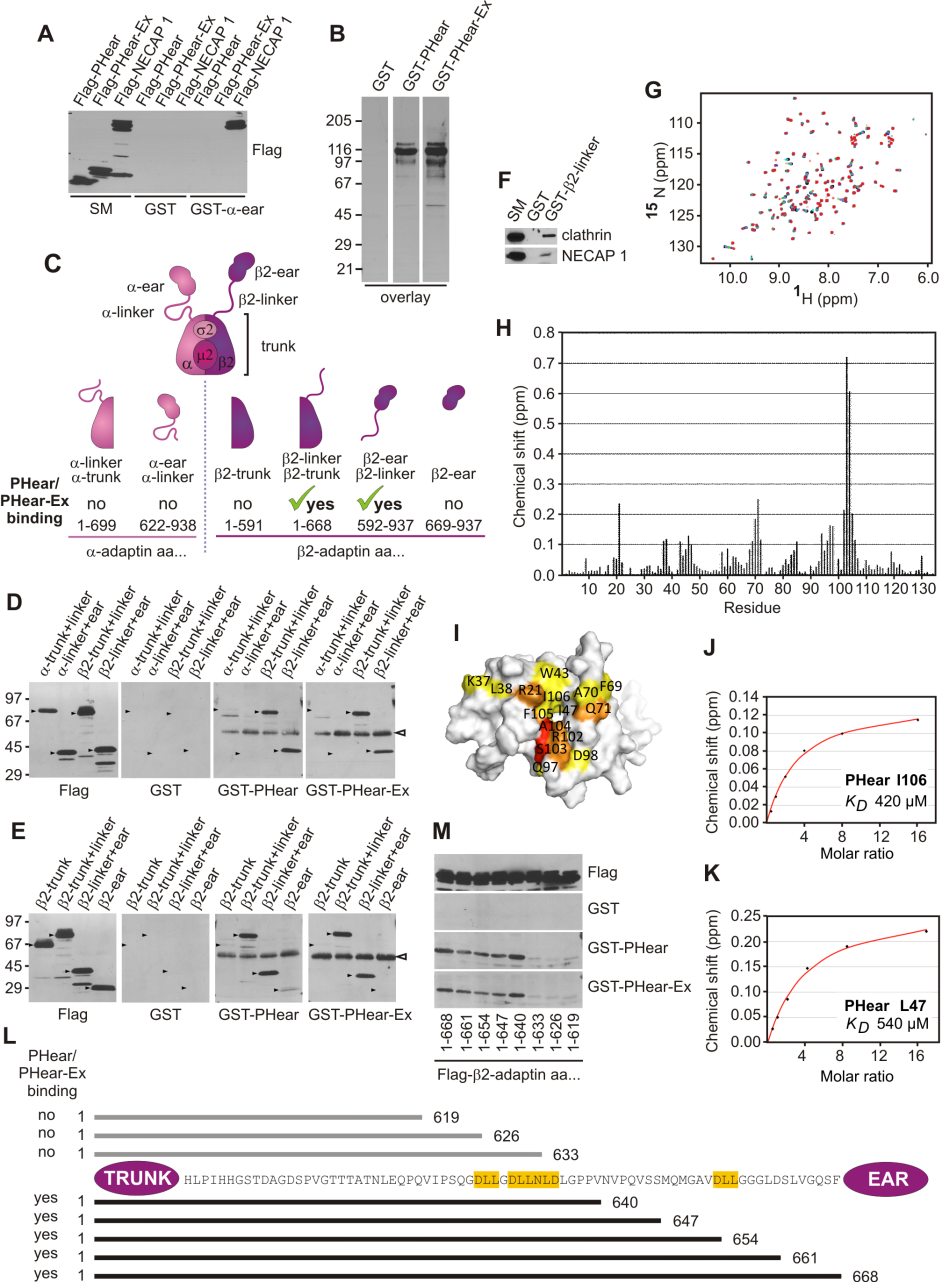
PHear domain binding to the AP-2 β2-linker. (A) Affinity selection of Flag-tagged PHear, PHear–Ex, and NECAP 1 from HEK-293–T cells using purified GST or GST fused to the α-ear of AP-2. (B) The coat protein fraction stripped from purified brain CCVs was overlaid with purified GST or GST fused to PHear or PHear–Ex (overlay). (C) Schematic representation of the heterotetrameric AP-2 complex (top) and deletion variants of the two large subunits α-adaptin and β2-adaptin. The amino acid (aa) positions indicate the borders of the respective construct, and their binding properties to PHear and PHear–Ex are indicated. (D and E) Flag-tagged variants of α- and β2-adaptin as indicated were immunoprecipitated from HEK-293–T cell lysates, and equal aliquots were resolved by SDS-PAGE to give four replicates. One was used for Western blotting with Flag antibody. The other three were overlaid with either purified GST or GST fused to PHear or PHear–Ex. The hollow arrowhead indicates the migratory position of the IgG heavy chain, detected on the blot. (F) Affinity selection of endogenous clathrin and NECAP 1 from brain lysate using purified GST or GST-β2-linker. (G) HSQC spectra showing NMR titrations of ^15^N-labeled PHear with the β2-linker peptide. (H) Magnitude of the amide chemical shift changes of NECAP 1 PHear residues upon binding of the β2-linker peptide. (I) Molecular surface representation of PHear. Amino acids implicated in β2-linker binding by NMR are labeled and colored. Color shading represents the size of the amide chemical shift changes (red, Δδ>0.3; orange, 0.3>Δδ>0.2; yellow, 0.2>Δδ>0.1 ppm). (J and K) The degree of chemical shift change (Δδ) determined by NMR of I106 (J) and L47 (K) in PHear is plotted against the concentration of the β2-linker peptide added. Red lines are lines of best fit. Calculated affinities (*K_D_*) are given for the peaks indicated. (L) Schematic representation of the AP-2 β2 subunit with a focus on the linker sequence. The trunk and ear regions are represented by circles at the N- and C-terminus, respectively. Peptide motifs for binding to the terminal domain of clathrin heavy chain (LLNLD and DLL) are highlighted in yellow. The bars indicate deletion variants of the β2-trunk+linker (aa 1–668). The numbers on each side represent the amino acid positions of the borders. Variants not binding to PHear and PHear–Ex are indicated by grey bars located above the amino acid sequence, while variants that do interact are indicated by black bars below the amino acid sequence. (M) Flag-tagged variants of β2-adaptin as indicated were immunoprecipitated from HEK-293–T cell lysates and equal aliquots were resolved by SDS-PAGE to give four replicates. One was used for Western blotting with Flag antibody. The other three were overlaid with either purified GST or GST fused to PHear or PHear–Ex. (A and F) Starting material (SM) represents 10% of the sample used in the binding assays.

To better understand the multiple PHear–Ex interactions, we first sought to identify the binding site(s) for PHear and PHear–Ex in AP-2. In affinity selection assays, GST-α-ear shows no binding to PHear or PHear–Ex, despite strong binding to full-length NECAP 1, which contains the C-terminal WxxF motif (Figure 2A). Coupled with the fact that NECAP 1 does not bind the AP-2 β2-ear [19], it becomes clear that PHear and Ex do not target the canonical AP-2 ear interfaces.

We next sought to identify the subunit of the AP-2 heterotetramer involved in PHear–Ex binding. Overlays (far-Western blots) provide a powerful means to identify and study protein–protein interactions. For example, synaptojanin was originally identified based on its interaction with the SH3 domains of Grb2 in overlay assays [22]. We thus performed overlay assays on coat proteins stripped from purified CCVs using soluble GST–PHear and GST–PHear–Ex, and both constructs revealed a strong signal at approximately 110 kDa, near the apparent MW of the two large AP-2 subunits α and β2 (Figure 2B). To distinguish between these subunits and further map the site, we immunoprecipitated Flag-tagged deletion variants of α and β2 containing either the trunk and linker or the linker and ear (Figure 2C) and used these in overlays. Both PHear and PHear–Ex interact with β2 variants with no binding to the α variants (Figure 2D). We next used variants containing β2 trunk alone, trunk and linker, linker and ear, and ear alone (Figure 2C). PHear and PHear–Ex only bind β2 variants containing the linker (Figure 2E). Throughout these experiments, the presence of Ex did not change the binding pattern of PHear–Ex compared to PHear. Unfortunately, we are unable to map the Ex binding site on AP-2 as Ex in isolation shows limited AP-2 binding (Figure 1D).

Mapping the site of PHear interaction to the β2-linker by overlay allowed us to confirm the interaction using in-solution affinity selection assays. GST–β2-linker specifically bound NECAP 1 from brain extracts with binding similar to that seen for clathrin, the only other protein known to interact with the β2-linker (Figure 2F). NMR analysis reveals that the β2-linker binds a site on PHear that overlaps with the interface for FxDxF motifs (Figure 2G–I) and titrations of β2-linker with PHear reveal a mean K_D_ of 480 µM (Figure 2J,K). While the affinity of PHear for the β2-linker is low in isolation, full-length NECAP 1 has two additional binding sites on AP-2 that provide avidity effects, the low affinity site in Ex and the high affinity WxxF motif at the C-terminus (Figure 1C).

Using C-terminal deletion variants of the β2 trunk and linker in overlay assays, we determined that critical residues for PHear binding are located in the linker between amino acids 633 and 640 (Figure 2L,M). Intriguingly, this area overlaps with the binding motif for the terminal domain of clathrin heavy chain (Figure 2L) [23]. Together, these data indicate that PHear binds the β2-linker of AP-2 and that clathrin and NECAP 1 may compete for access to the β2-linker.

**Figure 3:**
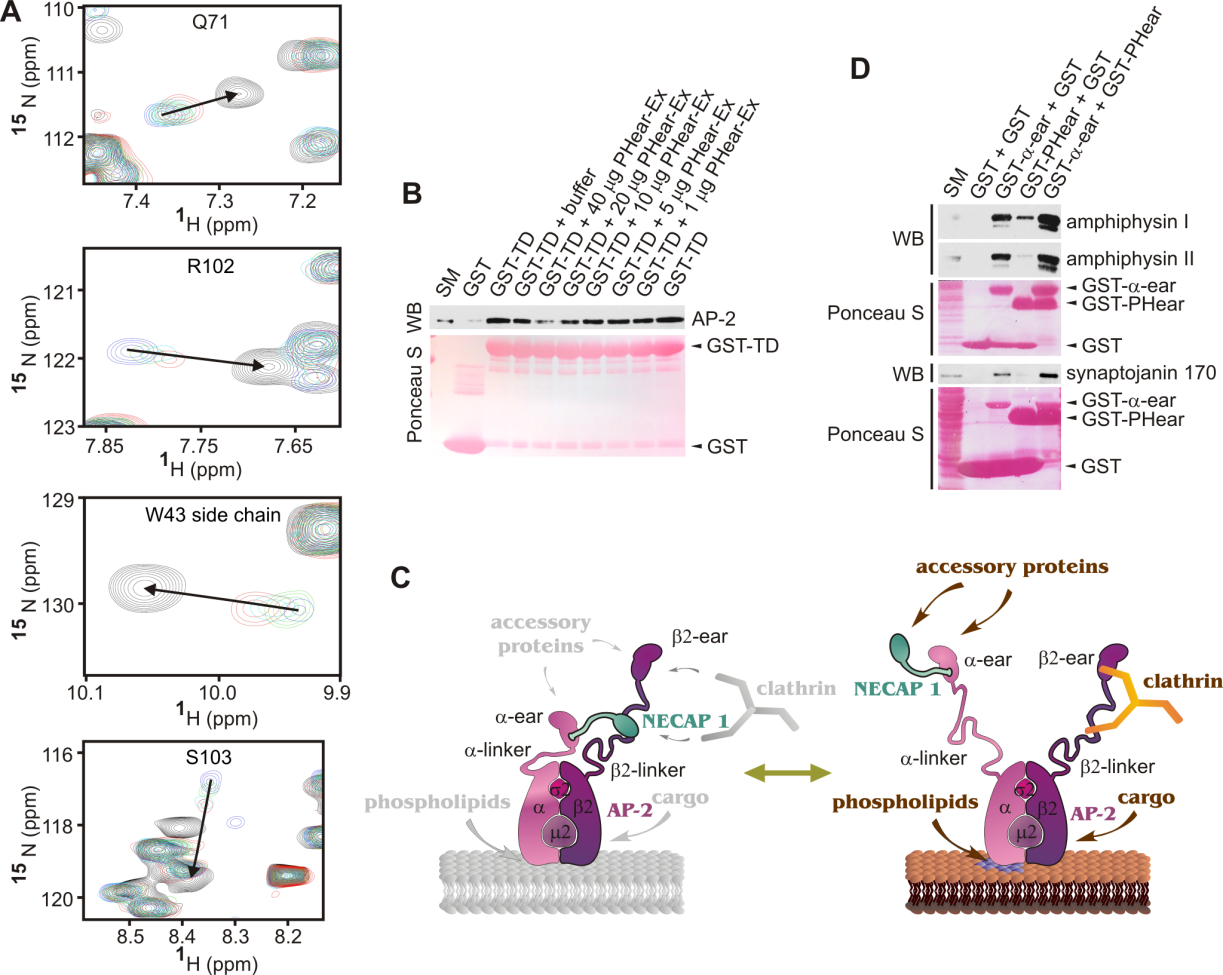
PHear competes with clathrin for the AP-2 β2-linker and cooperated with the AP-2 α-ear for accessory protein recruitment. (A) HSQC showing NMR titrations of selected residues as indicated of ^15^N-labeled PHear in complex with unlabeled β2-linker (blue) and titrated with unlabeled clathrin terminal domain (green, turquoise, red) in comparison to ^15^N-labeled PHear alone (black). Molar ratios of PHear∶β2-linker∶clathrin terminal domain for the various conditions tested are: black, 1∶0∶0; blue, 1∶4∶0; green, 1∶4∶0.5; turquoise, 1∶4∶1; red, 1∶4∶2. The arrows indicate the direction of shift towards the unbound position. (B) Whole AP-2 purified from brain CCVs was affinity selected using purified GST or the terminal domain of clathrin heavy chain fused to GST (GST-TD), and then some samples were incubated with either buffer alone or buffer containing various amounts of purified PHear–Ex as indicated. The Western blot (WB) reveals the amount of AP-2 bound to GST or GST-TD, and the Ponceau S stain of the membrane confirms even amounts of GST-TD throughout the assay. (C) Model of two NECAP 1/AP-2 complexes, one prior to vesicle formation (left) and the other involved in accessory protein recruitment during vesicle formation (right). The indicated proteins are not drawn to scale. (D) Affinity selection of α-ear and PHear binding partners from brain lysate using either GST alone, GST-α-ear and GST, GST-PHear and GST, or GST-α-ear and GST-PHear together. The WB reveals the amount of accessory proteins, as indicated, affinity selected in the various conditions. The Ponceau S staining of the membrane confirms constant amounts used for each fusion protein. (B and D) Starting material (SM) represents 10% of the sample used in the binding assays.

To test for competition between clathrin and NECAP 1 for AP-2 binding, we performed NMR studies with purified, ^15^N-labeled PHear in the presence of β2-linker and increasing concentrations of clathrin terminal domain. β2-linker causes chemical shift changes in PHear as it interacts with residues in the PHear binding site and these chemical shift changes are reversed to the ligand-free position in the presence of increasing concentrations of terminal domain (Figure 3A). We next performed more classical in-solution competition experiments. AP-2 was partially purified from stripped CCVs using gel filtration chromatography and bound to GST-clathrin terminal domain immobilized on Sepharose beads. Addition of increasing concentrations of purified PHear–Ex causes a dose-dependent decrease in the binding of AP-2 to clathrin terminal domain (Figure 3B), confirming that PHear–Ex and clathrin compete for binding to the β2-linker.

In summary, the data presented in Figures 1–3 indicate that NECAP 1 and AP-2 form two distinct complexes. In the first, the WxxF motif targets the α-ear, while PHear targets the β2-linker. This complex would likely be pre-endocytic because PHear occupies the clathrin-binding site in the β2-linker (Figure 3C). Once vesicle formation is initiated, clathrin competes PHear off the β2-linker, leading to the formation of the second complex, in which NECAP 1 remains bound to the sandwich domain of the AP-2 α-ear through the high-affinity WxxF motif, while PHear and the α-ear platform domain are positioned to cooperate in accessory protein recruitment (Figure 3C). Consistently, we detect cooperation of PHear and α-ear in binding to the FxDxF proteins amphiphysin I and II and synaptojanin 170 (binding is greater when the two domains are mixed than the sum of binding to the two domains on their own) (Figure 3D).

### Vesicle Size Is Increased and Vesicle Number Is Decreased Following NECAP 1 KD

**Figure 4:**
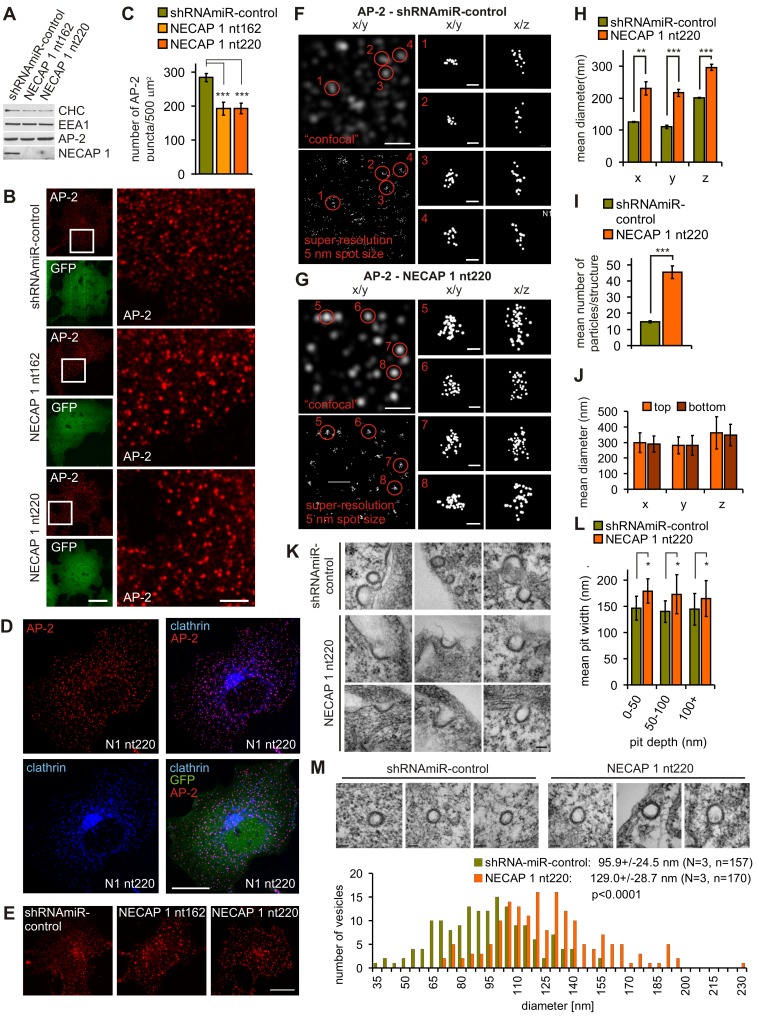
The number of vesicle formation sites and vesicle size are altered upon NECAP 1 KD. (A) Equal protein amounts of lysates from COS-7 cells transduced with control virus (shRNAmiR-control) or viruses to KD NECAP 1 (NECAP 1 nt162, NECAP 1 nt220) were processed by Western blot for the indicated proteins, CHC, clathrin heavy chain; EEA1, early endosomal antigen 1. (B) Immunofluorescence analysis of endogenous AP-2 in control and NECAP 1 KD COS-7 cells. The large panel is a magnification of the area boxed in the small AP-2 panel. The bar represents 20 µm in the small and 5 µm in large panels. (C) Quantification of (B). Repeated measures one-way ANOVA followed by Bonferroni's Multiple Comparison Test revealed a significant difference between control and KD cells, ***p<0.0001, *N* = 4. (D) NECAP 1 KD COS-7 cells were processed for immunofluorescence to reveal endogenous AP-2 and clathrin light chains (clathrin). The bar represents 20 µm. In (B and D), GFP is expressed as part of the viral expression cassette to verify transduction. (E) Distribution of Cy3-labeled transferrin endocytosed for 1 min in control (shRNAmiR-control) and NECAP 1 KD (NECAP 1 nt162 and NECAP 1 nt220) COS-7 cells. The bar represents 20 µm. (F and G) 3D superresolution analysis of AP-2-labeled vesicle formation sites in control (F) and NECAP 1 KD cells (G). The large panels show a 4 µm^2^ area with biplane signals rendered to a 250 nm particle size (“confocal”) to illustrate a traditional microscope image, and rendered to a 5 nm particle size (superresolution). The small panels show individual structures from the large panels as indicated in x/y and x/z orientation with signals rendered to a 5 nm particle size. The 5 nm spot size exceeds the localization precision of each point but shows the distribution and numbers of localizations. The scale bar represents 1 µm in the large and 100 nm in the small panels. (H) Quantification of the x, y, and z diameters of deeply invaginated CCPs in control and NECAP 1 KD cells. Unpaired two-tailed *t* tests revealed significant differences in size for all three dimensions, *x*-axis, **p = 0.0038; *y*-axis, ***p<0.0001; *z*-axis, ***p<0.0001; shRNAmiR-control: four cells, 40 vesicle formation sites; NECAP 1 nt 220: six cells, 60 vesicle formation sites. (I) Quantification of the number of AP-2 signals detected per vesicle formation site. An unpaired two-tailed *t* test revealed a significant difference, ***p = 0.0002; shRNAmiR-control: four cells, 40 vesicle formation sites; NECAP 1 nt 220: six cells, 60 vesicle formation sites. (J) Quantification of the x, y, and z diameters of deeply invaginated vesicle formation sites at the bottom (cell surface contacting the cover slip) or top (cell surface facing the culture medium) of NECAP 1 KD cells. Unpaired two-tailed *t* tests revealed no significant differences in size for all three dimensions, each location: three cells, 23 vesicle formation sites. (K and L) Size analysis of CCPs in control and NECAP 1 KD COS-7 cells measuring the width of CCPs detected by EM. (K) The electron micrographs show representative examples of structures found in control and KD cells. The bar equals 100 nm. (L) The bar graph shows the average width of CCPs binned for pits depths of 0–50 nm, 50–100 nm, and 100+ nm in control and NECAP 1 KD cells. Statistical analysis using a two-tailed Mann Whitney test revealed a significant difference in size between the two populations in each bin, 0–50 nm, *p = 0.0286; 50–100 nm, *p = 0.0370; 100+, *p = 0.0280. (M) Size analysis of CCVs in control and NECAP 1 KD COS-7 cells measuring the membrane-to-membrane diameter of CCVs detected by EM. The electron micrographs show representative examples of structures found in control and KD cells (bars equal 100 nm), and the bar graph shows the size distribution within the whole population of vesicles analyzed in each condition. Statistical analysis using a two-tailed unpaired *t* test revealed a significant difference in size between the two populations, ***p<0.0001, *N* = 3.

To address which steps of vesicle formation are dependent on the cooperation of NECAP 1 and AP-2, we knocked down NECAP 1 in COS-7 cells (Figure 4A), which express NECAP 1 at levels similar to other cultured cell lines (Figure S1) and tested for changes in vesicle formation at the plasma membrane. Two obvious alterations were observed following NECAP 1 KD: (1) a reduction in the number of AP-2 puncta at the plasma membrane (Figure 4B,C) and (2) an increase in AP-2 signal intensity in a large proportion of the remaining AP-2 puncta (Figure 4B). These AP-2 structures still co-localize with clathrin (Figure 4D), suggesting that they are functional vesicle formation sites. Endocytic vesicles labeled with transferrin following 1 min of uptake are also fewer in number and brighter following NECAP 1 KD (Figure 4E), indicating that the changes occurring at the level of the plasma membrane are maintained in the early endocytic pathway.

There is a direct correlation between the immunofluorescence signal intensity of coat proteins such as AP-2 and clathrin and the size of the forming structure [24]–[26]. To examine if the increase in AP-2 signal intensity reflects an increase in the size of vesicle formation sites, we used 3D superresolution microscopy (Figure 4F–J, Figures S2 and S3, and Movies S1, S2, S3, S4). Quantification of the diameters of deeply invaginated CCPs in x, y, and z confirms that NECAP 1 KD causes a size increase in all three dimensions (Figure 4F–H). There is also an increase in the number of AP-2 signals detected per vesicle formation site (Figure 4F,G,I), confirming that the increase in AP-2 signal intensity seen by confocal microscopy serves as a reliable readout of increased size. In addition to CCPs, some cell lines form large planar clathrin-coated plaques but only at the membrane opposed to the glass surface (bottom) [27]. To address whether NECAP 1 KD leads to an increase in CCP size or to a shift towards coated plaques, we compared the dimensions of AP-2-labeled structures on the membrane contacting the coverslip (bottom) and on the cell surface facing the culture medium (top). Both locations show the same three-dimensional increase in diameter (Figure 4J, Figure S3), indicating that NECAP 1 is needed to control the size of CCPs. To further validate this result, we performed EM analysis (Figure 4K,L). We measured the depth of the pits and placed them into three bins of 0–50 nm, 50–100 nm, and 100+ nm depth. In all three bins, the mean width of the pits was significantly increased in the NECAP 1 KD cells (Figure 4L). Notably, we found no evidence for clusters of CCPs, indicating that NECAP 1 KD causes an increase in pit diameter and not an increased clustering of pits.

We next used EM to investigate if the increase in the size of vesicle formation sites translates into larger CCVs. Indeed, the absence of NECAP 1 causes a population-wide shift towards larger CCVs (Figure 4M). The EM data also confirm that despite the increase in size, the clathrin-coated structures still complete the vesicle formation process (Figure 4M).

### Synaptic Vesicle Size Is Increased Upon NECAP 1 KD

Controlling the size and number of CCVs is critical in all cell systems but nowhere more so than in the presynaptic nerve terminal. Synaptic vesicles are the smallest known transport vesicles and are reformed by clathrin-mediated endocytosis following neurotransmitter release [28]–[32]. Size control is especially important for synaptic vesicles as their size determines the neurotransmitter content [33]. NECAP 1 is expressed at highest levels in brain and is enriched in purified CCVs and synaptic vesicles [19],[34]. KD of NECAP 1 in primary hippocampal neurons leads to a reduction in synaptic vesicle number and an average increase of 12–14% in synaptic vesicle diameter (Figure S4), which translates to a change in vesicle volume of nearly 50%. Thus, NECAP 1 serves a similar function in the endocytic machineries of nonneuronal cells and neurons. We assume that the effects on vesicle number and size following NECAP 1 KD are indirect as NECAP 1 functions together with AP-2 to coordinate the recruitment of endocytic accessory proteins during vesicle formation.

### NECAP 1 Bridges Accessory Proteins to AP-2 for Efficient Vesicle Formation

**Figure 5:**
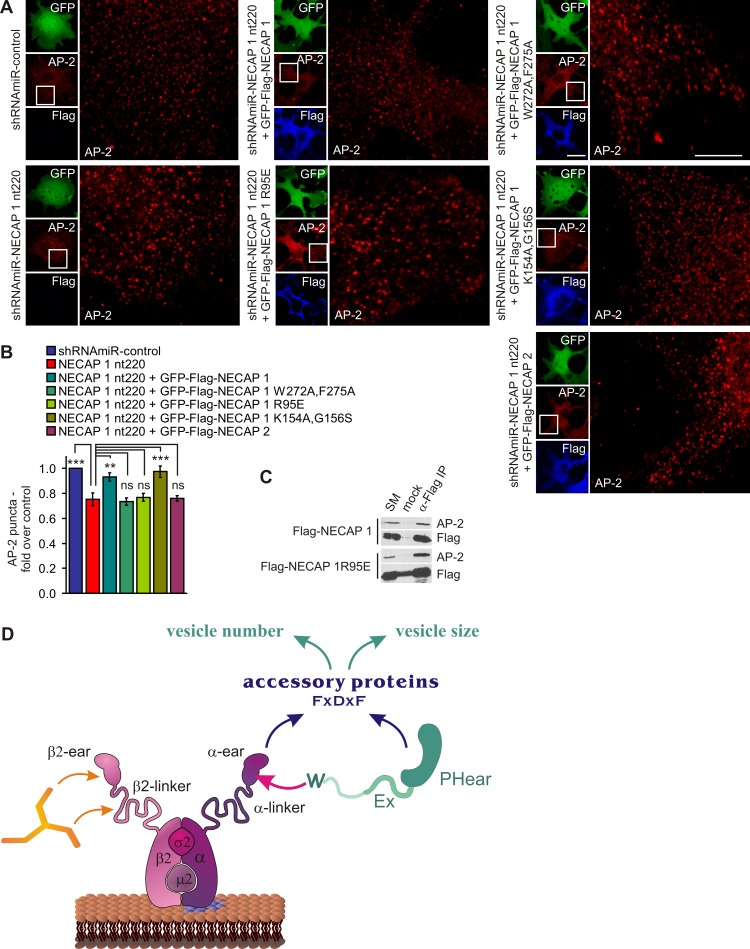
Essential NECAP 1 interactions for efficient vesicle formation. (A) Immunofluorescence analysis of endogenous AP-2 in control (shRNAmiR-control) and NECAP 1 KD (NECAP 1 nt220) COS-7 cells. In some cases as indicated, NECAP 1 KD cells were transduced to express GFP-Flag-tagged NECAP 1 variants. The right panels are magnifications of the boxed areas. The bars represent 20 µm in the small and 5 µm in the large panels. (B) Quantification of AP-2 puncta per cell with control virus set to 1. Statistical differences for some NECAP 1 variants compared to NECAP 1 KD cells were detected by repeated measures one-way ANOVA followed by Dunnett's Multiple Comparison Test to compare NECAP 1 nt220 to all other conditions, **p<0.01, ***p<0.001, ns = nonsignificant, *N* = 3. (C) Co-immunoprecipitation of endogenous AP-2 from HEK-293–T cells transfected with Flag-tagged NECAP 1 variants as indicated using Flag antibody. Starting material (SM) represents 10% of the protein amount used in each binding. (D) Model indicating the interactions of NECAP 1 and AP-2 contributing to efficient accessory protein recruitment.

To determine how NECAP 1 is involved in controlling the number and size of CCVs, we performed rescue experiments with NECAP 1 wild-type and point mutants. One essential feature of NECAP 1 is its ability to interact with the AP-2 α-ear and a NECAP 1 variant in which the high affinity C-terminal WxxF motif is inactivated fails to rescue the NECAP 1 KD phenotypes (Figure 5A,B). In contrast, re-expression of wild-type NECAP 1 restores the number of vesicle formation sites (Figure 5A,B) and also leads to lower AP-2 intensity levels in these structures. To address the importance of PHear-mediated interactions, we tested a mutant (R95E) that disrupts PHear binding to FxDxF motifs and the AP-2 β2-linker (Figure 1H). This mutant fails to rescue the NECAP 1 KD phenotype (Figure 5A,B) despite its ability to target the AP-2 α-ear as seen by its co-immunoprecipitation with AP-2 (Figure 5C). A NECAP 1 mutant in which the AP-2-binding site in Ex was mutated (K154A/G156S) (Figure 1G) rescues the phenotype (Figure 5A,B). This suggests that the ability of Ex to enhance AP-2 interactions with the NECAP 1 N-terminus is dispensable during vesicle formation. Within full-length NECAP 1, Ex has by far the lowest affinity to AP-2 and if at all, may only play a role in pre-endocytic NECAP 1/AP-2 interactions. Finally, expression of wild-type NECAP 2 [19] does not rescue the NECAP 1 KD phenotype (Figure 5A,B), demonstrating that the two mammalian NECAP isoforms are functionally divergent. This is consistent with our observations that NECAP 2 is not involved in clathrin-mediated endocytosis but instead functions in endosomal sorting (our unpublished data).

### NECAP 1 Recruits CALM to Control Vesicle Size and Cargo Content

**Figure 6:**
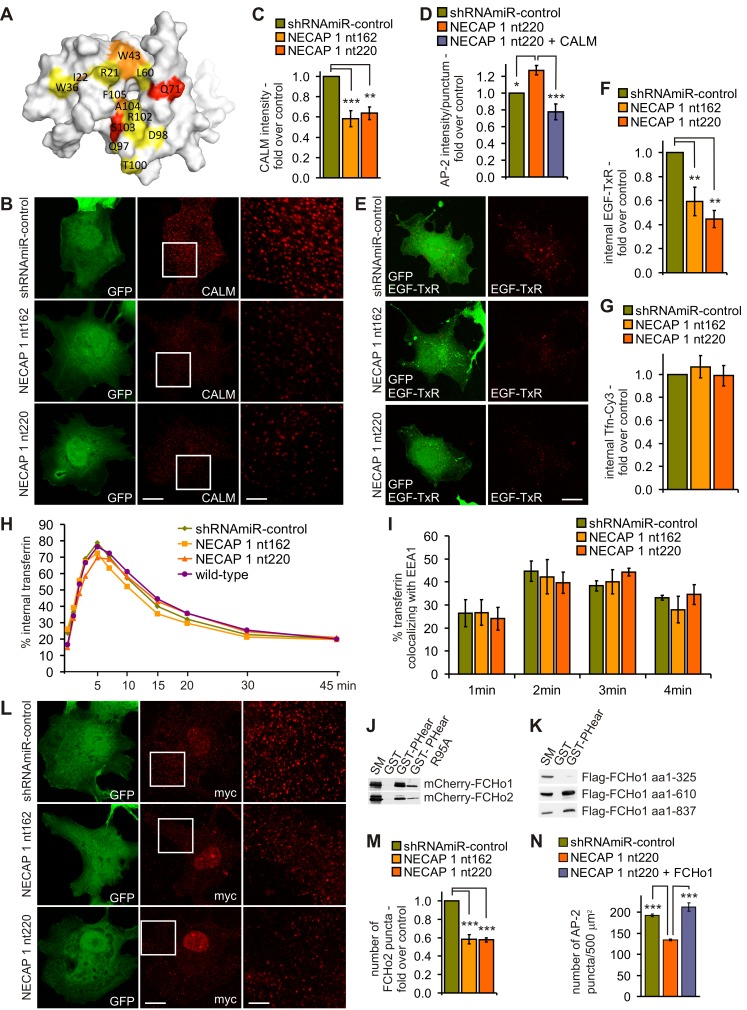
NECAP 1 controls accessory protein levels during vesicle formation, which in turn regulate vesicle number, size, and cargo. (A) Molecular surface representation of PHear. Amino acids implicated in AP180 binding by NMR are labeled and colored according to the size of the amide chemical shift changes observed upon ligand binding (red, Δδ>0.3; orange, 0.3>Δδ>0.2; yellow, 0.2>Δδ>0.1 ppm). (B) Immunofluorescence analysis of endogenous CALM in control and NECAP 1 KD COS-7 cells. GFP is expressed as part of the viral expression cassette to verify transduction. The large panel is a magnification of the area boxed in the small CALM panel. The bar represents 15 µm in the small and 5 µm in large panels. (C) Quantification of CALM intensity per punctum detected in (B). Repeated measures one-way ANOVA followed by Bonferroni's Multiple Comparison Test revealed a significant difference between control and KD cells, **p<0.01, ***p = 0.0008, *N* = 3. (D) Quantification of AP-2 intensity per vesicle formation site in control, NECAP 1 KD cells, and NECAP 1 KD cells expressing mCherry-tagged CALM. Repeated measures one-way ANOVA followed by Dunnett's Multiple Comparison Test revealed a significant difference between control and KD cells and between KD cells and KD cells expressing CALM, *p<0.05, ***p = 0.0004, *N* = 6. (E) Immunofluorescence analysis of Texas Red–labeled EGF endocytosis over 2.5 min in control and NECAP 1 KD COS-7 cells. GFP is expressed as part of the viral expression cassette to verify transduction. The bar represents 15 µm. (F) Quantification of EGF endocytosis in control and NECAP 1 KD COS-7 cells over 2.5 min presented in (E). Repeated measures one-way ANOVA followed by Bonferroni's Multiple Comparison Test revealed significant differences between control and NECAP 1 KD cells, **p = 0.0017, *N* = 4. (G) Quantification of transferrin endocytosis in control and NECAP 1 KD COS-7 cells over 2.5 min. Repeated measures one-way ANOVA followed by Bonferroni's Multiple Comparison Test revealed no significant differences, *N* = 5. (H) FACS analysis of transferrin endocytosis and recycling in control and NECAP 1 KD COS-7 cells alongside nontransduced wild-type COS-7 cells. Repeated measures two-way ANOVA followed by Bonferroni posttests revealed no significant differences, *N* = 4. (I) Quantification of the immunofluorescence analysis of the overlap of endogenous EEA1 and Cy3-labeled transferrin endocytosed in control and NECAP 1 KD COS-7 cells at the time points indicated. Repeated measures two-way ANOVA followed by Bonferroni posttests revealed no significant differences, *N* = 4. (J) Affinity selection of mCherry-tagged FCHo1 and 2 from HEK-293–T cells using purified GST or GST fused to PHear or PHear R95A. (K) Affinity selection of Flag-tagged FCHo1 variants (aa1–327, F-BAR-x alone; aa1–610, F-BAR-x+linker; aa1–837, full-length; aa, amino acids) from HEK-293–T cells using purified GST or GST fused to PHear. (J and K) Starting material (SM) represents 10% of the lysate used in the binding assays. (L) Immunofluorescence analysis of myc-tagged FCHo2 in control and NECAP 1 KD COS-7 cells. GFP is expressed as part of the viral expression cassette to verify transduction. Endogenous myc is detected in the nuclei. The large panel is a magnification of the area boxed in the small myc panel. The bar represents 15 µm in the small and 5 µm in large panels. (M) Quantification of the number of myc-FCHo2 puncta detected in (L). Repeated measures one-way ANOVA followed by Bonferroni's Multiple Comparison Test revealed a significant difference between control and KD cells, ** *p* = 0.0014, *N* = 3. (N) Quantification of number of AP-2 puncta per 500 µm^2^ in control, NECAP 1 KD cells, and NECAP 1 KD cells expressing mCherry-tagged FCHo1. Repeated measures one-way ANOVA followed by Dunnett's Multiple Comparison Test revealed a significant difference between control and KD cells and between KD cells and KD cells expressing FCHo1, ***p<0.001, *N* = 6.

Our findings indicate that NECAP 1 functions to modulate the ability of AP-2 to recruit accessory proteins during vesicle formation. We thus set out to determine which accessory proteins might be responsible for the alterations in vesicle size and number seen following NECAP 1 KD. In respect to vesicle size, we tested for an effect of NECAP 1 KD on AP180/CALM. CALM is a clathrin- and AP-2-binding endocytic protein, and the increase in vesicle size in NECAP 1 KD cells is reminiscent of a phenotype observed in CALM KD cells [35]. Similarly, functional disruption of the neuronal isoform of CALM, AP180 leads to an increase in the size of synaptic vesicles [36]–[40]. Both CALM and AP180 contain FxDxF motifs that interact with NECAP 1 [21], and NMR binding studies demonstrate that an FxDxF-motif peptide derived from AP180 binds to the canonical interface on PHear (Figure 6A). The increase in vesicle size following NECAP 1 KD could thus result from reduced levels of CALM [35] and indeed, in the absence of NECAP 1, less CALM is observed at sites of vesicle formation (Figure 6B,C). Overexpression of CALM in NECAP 1 KD cells could provide a means to increase CALM at these sites and thus allow the endocytic machinery to rebalance. Indeed, CALM expression in the absence of NECAP 1 led to a decrease in the size of vesicle formation sites as judged by AP-2 intensity (Figure 6D). Thus, NECAP 1 is required for efficient recruitment of CALM to vesicle formation sites where CALM regulates vesicle size, either directly or indirectly.

CALM also plays a role in cargo selection by CCPs, even though the cargo-specific effects remain poorly understood. For example, CALM serves as a cargo adapter for R-SNAREs [40]–[42]. In addition, KD of CALM reduces clathrin-dependent endocytosis of amyloid precursor protein and EGF receptor without influencing transferrin receptor endocytosis [43],[44]. Given that NECAP 1 KD reduces the level of CALM recruited to forming vesicles, we hypothesized that NECAP 1 KD would lead to a selective disruption of cargo entry. Indeed, NECAP 1 KD decreases the clathrin-dependent internalization of EGF by over 40% (Figure 6E,F), similar to the reduction in EGF internalization seen upon CALM KD [44], while transferrin endocytosis and recycling was not altered (Figure 6G–I). The changes in vesicle size and cargo we observe upon NECAP 1 KD are thus likely a result of the reduced levels of AP180/CALM recruited during vesicle formation.

### NECAP 1 Bridges AP-2 to the FCHo Complex

To better understand how NECAP 1 KD causes a decrease in vesicle number, we tested for effects on the FCHo complex. The members of the FCHo protein family form tri-partite complexes with the endocytic accessory proteins Eps15 and intersectin [7],[8]. FCHo expression levels directly correlate with the number of vesicle formation sites and successful endocytic events, while destabilization of FCHo complexes interferes with vesicle formation [8],[9]. Interestingly, both FCHo1 and 2 interact with PHear and this binding is reduced in the PHear variant R95A (Figure 6J), which also shows reduced binding to FxDxF proteins and AP-2 (Figure 1H). Testing a range of deletion variants revealed that PHear binds the central linker region of FCHo1 (Figure 6K), which is flanked by the N-terminal FCH domain and the C-terminal mu homology domain. NECAP 1 thus provides a parallel mechanism to Eps15 to interlink the FCHo complex with AP-2, helping to stabilize and maintain normal numbers of vesicle formation sites. Given the interaction between NECAP 1 and FCHo1/2, we tested for an effect of NECAP 1 KD on the FCHo complex using FCHo2 as a marker. NECAP 1 deletion causes a decrease in the number of FCHo2 puncta at the plasma membrane (Figure 6L,M), revealing that NECAP 1 is needed to efficiently stabilize the FCHo complex at the membrane. Given the correlation between FCHo expression levels and number of vesicle formation sites [8],[9], we reasoned that FCHo overexpression would increase the number of formation sites in NECAP 1 KD cells. Indeed, overexpression of FCHo1 in NECAP 1 KD cells efficiently increased the number of AP-2 puncta at the plasma membrane (Figure 6N).

Together, these data demonstrate that the cooperation of NECAP 1 and AP-2 allows for efficient protein recruitment into the endocytic protein network to control important aspects of CCV formation such as vesicle number, cargo content, and size.

## Discussion

Most proteins of the endocytic machinery are categorized as accessory proteins. In general, accessory proteins are recruited to sites of vesicle formation in a temporally controlled manner to facilitate specific steps—for example, the FCHo complex and/or clathrin/AP-2 [7]–[9] initiate vesicle formation at PI(4,5)P_2_-rich spots on the plasma membrane, epsin and clathrin are involved in membrane deformation during invagination, and a late burst of dynamin recruitment is required for vesicle scission [2]. As such, efficient vesicle formation depends on recruiting the correct set of accessory proteins in sufficient amounts at specific times. However, we have only rudimentary insights into how recruitment of accessory proteins is coordinated and regulated during CCV formation.

**Figure 7:**
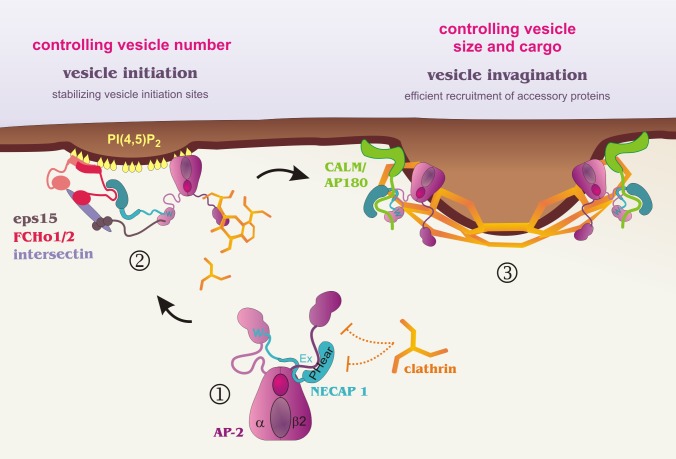
Model of vesicle formation. (Step 1) The NECAP 1 WxxF motif targets the α-ear, while PHear–Ex engages the β2-linker (and the unknown area targeted by Ex), creating a pre-endocytic NECAP 1/AP-2 complex and preventing premature clathrin recruitment. The NECAP 1/AP-2 complex then scans PI(4,5)P_2_-enriched patches at the plasma membrane for suitable vesicle initiation sites. (Step 2) The trimeric FCHo/Eps15/intersectin complex concentrates in PI(4,5)P_2_-rich patches. Binding of the α-ear to Eps15 (DPF and FxDxF motifs) and PHear domain binding to Eps15 and FCHo1 and 2 interlock the FCHo1 and AP-2/clathrin protein complexes to stabilize vesicle initiation sites. Clathrin recruitment by AP-2 could promote these interactions by competing PHear off the β2-linker, allowing PHear/α-ear to cooperate in accessory protein binding. (Step 3) During vesicle formation, AP-2 and NECAP 1 cooperate to recruit FxDxF motif-containing accessory proteins such as CALM and AP180. The amount of these accessory proteins recruited to the forming vesicle in turn determines the size of the vesicle, and in the case of CALM also regulate the incorporation of cargo such as EGF receptor.

AP-2 is a central hub of the endocytic machinery, coordinating the recruitment of clathrin, cargo, and numerous accessory proteins. The data presented here identify NECAP 1 as a modulator of AP-2 interactions (Figure 7). NECAP 1 engages the sandwich domain of the AP-2 α-ear through its C-terminal WxxF motif, while PHear engages the β2-linker at a site overlapping with the binding site for the terminal domain of clathrin. Engagement of the β2-linker by clathrin frees PHear for synergistic interactions along with the platform part of the α-ear for recruitment of endocytic accessory proteins (Figure 7).

Recent studies with endogenously tagged proteins suggest that cargo recognition is not required to stabilize vesicle formation sites [45]. Therefore, the rate and efficiency of vesicle initiation is the major and perhaps only step that controls the endocytic capacity of a cell. However, the low number of molecules involved still hampers our ability to directly study these early events of vesicle formation in great detail. In one model of vesicle initiation, AP-2 stochastically samples the plasma membrane for PI(4,5)P_2_-rich sites, where it coordinates the recruitment of other endocytic proteins [9]. An alternative model [8] favors the idea that vesicle initiation sites are determined by the recruitment of the FCHo complex to PI(4,5)P_2_-positive sites at the plasma membrane, with subsequent incorporation of AP-2 and clathrin. If one considers vesicle initiation as a short temporal window of opportunity in which the stochastic association of AP-2 and the FCHo complex with PI(4,5)P_2_ brings these factors together to allow for their interaction, the success rate of vesicle formation would depend on stabilizing these factors at the membrane long enough to nucleate the formation of the endocytic protein network. NECAP 1 is ideally positioned to accomplish this stabilization, and our data demonstrate that NECAP 1 is required to maintain normal numbers of vesicle formation sites. When the NECAP 1/AP-2 complex is recruited to vesicle initiation sites, the NECAP 1 PHear can release from the β2-linker and cooperate with AP-2 to engage the FCHo complex in a manner parallel to that of Eps15, thereby increasing the probability of coincidence detection that triggers vesicle formation (Figure 7). It is tempting to speculate that recruitment of clathrin to initiation sites functions as the switch to trigger vesicle formation, given that the clathrin terminal domain competes PHear off the β2-linker, thereby promoting the cooperation of PHear and α-ear in FxDxF protein binding. Concomitantly, the β2-ear and linker are now fully accessible and can recruit and polymerize clathrin at the site of vesicle formation, providing a scaffold for the growing protein network. The fact that FCHo overexpression in NECAP 1 KD cells overcomes the KD phenotype and rebalances vesicle numbers further attests to the necessity of stabilization factors such as NECAP 1 to concentrate accessory proteins in amounts sufficient for efficient vesicle formation.

The composition of the endocytic machinery also controls cargo recruitment. Some cargo is recognized by alternate adaptors, accessory proteins with the ability to link their cargo to AP-2 and clathrin [1]. Interestingly, the endocytic machinery also has an inherent ability to translate cargo content into vesicle size adaptation—for example, vesicles that internalize ligand-bound low-density lipoprotein receptors or viruses increase in size to harbor such large cargo [2],[24]. On the other hand, synaptic vesicles are recycled such that their characteristic small size is maintained. For neuronal transmission, this is important for two reasons: first, smaller vesicles can be formed faster and this might be needed to maintain synaptic vesicle pools during times of high neuronal activity. Second, the size of a vesicle directly determines vesicle volume, which in turn determines the quantal amount of neurotransmitter, with implications for the strength of synaptic transmission and synaptic potentiation [46]. Consistently, alterations in synaptic vesicle size have been demonstrated to cause changes in neurotransmission in nonmammalian and mammalian systems alike [39],[44],[46],[47]. The predominantly neuronal expression pattern of NECAP 1, together with the fact that NECAP 2 does not function in clathrin-mediated endocytosis, suggests that a tightly controlled and efficient recruitment of accessory proteins may be most critical at the synapse. However, the molecular mechanisms that determine vesicle size and couple cargo to size regulation remain elusive.

In nonneuronal cells, deletion of CALM causes an increase in vesicle size [35]. Similarly, deletion or mutation of the neuronal isoform AP180 leads to larger synaptic vesicles [36]–[40], demonstrating the importance of these accessory proteins for vesicle size control even though the precise mechanism of their function remains unclear. Both proteins also have cargo-specific effects, AP180 and CALM recruit synaptobrevin during synaptic vesicle recycling, and CALM also promotes EGF receptor and amyloid precursor protein internalization in nonneuronal cells [38],[40],[43],[44],[47]. Our study reveals that the cooperation of AP-2 and NECAP 1 is crucial for efficient CALM recruitment and suggests that decreased CALM levels due to NECAP 1 ablation cause the formation of larger vesicles. This is confirmed by the fact that CALM overexpression in NECAP 1 KD reverses the phenotype and allows for the formation of small vesicle formation sites in the absence of NECAP 1. It remains formally possible that the size increase in vesicle formation sites observed following NECAP 1 KD results from clustering of vesicle formation sites or an increase in endocytic hot spots [48], but our EM analysis of vesicle formation sites does not support these possibilities.

Live-cell imaging reveals virtually identical recruitment profiles of CALM and NECAP 1 during vesicle formation [49], strongly supporting the idea that NECAP 1 and CALM share a common function. Moreover, NECAP 1 KD specifically reduces clathrin-dependent endocytosis of EGF receptor with no effect on transferrin receptor internalization, reminiscent of the phenotypes specific for CALM KD [44]. Together, these data demonstrate the importance of NECAP 1 for efficient accessory protein recruitment to sites of vesicle formation and reveal how balancing the levels of endocytic proteins during vesicle formation in turn controls vesicle size and cargo selection.

## Materials and Methods

### Antibodies and Reagents

Mouse monoclonal antibodies against CHC (clone 23), α-adaptin (clone 8, for Western blotting), γ-adaptin (clone 88), and EEA1 (clone 14) were from BD Transduction Laboratories (Lexington, KY). Mouse monoclonals against α-adaptin (AP.6, for immunofluorescence) and Flag (M2) were from Thermo Scientific and Sigma (St-Louis, MO), respectively. Rabbit polyclonal antibody against c-myc (A-14) and goat polyclonal antibody against CALM (C-18) were from Santa Cruz and polyclonal antibodies against amphiphysin I/II (1874), clathrin light chains, and NECAP 1 and 2 have been described previously [19],[20],[50],[51]. The antibody against synaptojanin 170 was a generous gift of Dr. Pietro De Camilli (Yale University). AlexaFluor 633–conjugated human transferrin (T-23362) and biotinylated EGF complexed to Texas Red-Streptavidin (E-3480) were from Invitrogen and Cy3-conjugated human transferrin (009-160-050) was from Jackson ImmunoResearch (West Grove, PA). The mCherry tag was detected by Western blot using a mouse monoclonal antibody against RFP (abcam, Cambridge, MA, ab65856). The synthetic amphiphysin I peptide was purchased from the HHMI/Keck Biotechnology Resource Laboratory, Yale University. The synthetic peptides for AP180 and the β2-linker were purchased from Sheldon Biotechnology Center at McGill University.

### Constructs

The following constructs were described previously: GST–NECAP 1, GST–α-ear, and Flag–PHear–Ex (termed aa1–178) [19], Flag–NECAP 1 and Flag–NECAP 1 W272A, F275A (termed AVQA) [15], GST–PHear, GST–PHear–Ex (termed aa1–178), GST–Ex (termed aa129–178), and the GST–PHear point mutants [21]. cDNA clones for NECAP 1 (gi:27229051), NECAP 2 (gi:13384759), α-adaptin (gi:163644276), β2-adaptin (gi:71773037), FCHo1 (gi:255683302), and FCHo2 (gi:30854355) were used as PCR templates, and point mutations as needed were introduced using the megaprimer procedure [52]. For bacterial expression, inserts were subcloned into pGEX-4T1 or pGEX-6P1. For mammalian expression, inserts were subcloned into pcDNA3 with integrated Flag- or myc-tags (described in [20],[53]). For NMR studies using purified Ex, a PCR-amplified DNA fragment encoding amino acids 128–178 was subcloned into pPROEX-HTb for the expression of N-terminally His-tagged protein. For NECAP 1 KD, NECAP 1–specific target sequences for human and rat protein were designed using the Block-iT RNAi Designer (Invitrogen) and subcloned into pcDNA6.2/GW-EmGFP-miR (Invitrogen) following the manufacturer's instructions. The EmGFP-miR cassette was then amplified by PCR and subcloned into the pRRLsinPPT vector to generate the microRNA expression vectors. The number given in the name of each KD virus corresponds to the first nucleotide position targeted in the mRNA. The control virus has been described previously [54]. For generation of rescue/protein expression viruses, the microRNA part of the EmGFP expression cassette in pRRLsinPPT was replaced by a polylinker, which was subsequently used to clone Flag-tagged variants of NECAP 1 and 2 in frame with EmGFP such that EmGFP–Flag–NECAP fusion proteins were expressed. Expression vectors for mCherry-tagged FCHo1 and 2 were from addgene (Cambridge, MA, plasmids 27690 and 27686, respectively). The construct for GST-clathrin terminal domain was a gift of Dr. James Keen (Thomas Jefferson University, Philadelphia, PA).

### Cell Culture

HEK-293-T and COS-7 cells were maintained in DMEM High Glucose (Invitrogen) containing 10% FBS (PAA Laboratories Inc.), 100 U/ml penicillin, and 100 µg/ml streptomycin (both Invitrogen).

### Virus Production

For expression of VSVG pseudotyped virus, HEK-293-T were seeded with 10^7^ cells/plate on 15 cm plates in 25 ml of regular culture medium, with six plates for each virus. The following day, each microRNA or protein expression vector was co-transfected with a packaging mix (containing pMD2.g, pRSV-Rev, and pMDLg/pRRE, Addgene) using calcium phosphate. After 8 h, the medium was removed from each plate and replaced with 15 ml of collection medium per plate (regular medium supplemented with 1× nonessential amino acids (Gibco) and 1 mM sodium pyruvate (Gibco)). At 24, 36, and 48 h posttransfection, the medium was removed from each plate and for the 24 and 36 h time point, replaced with 15 ml collection medium. The supernatants for each construct and each collection were combined and stored at 4°C until the end of the collection procedure. The supernatants were then filtered through a 0.45 µm PES membrane and the virus was concentrated by centrifugation (8 h at 17,000× *g*), and the resulting pellets were resuspended in DMEM in 1/2,000 of the original volume. Concentrated virus was stored at −80°C until use. To determine the virus titer, HEK-293–T cells were plated in 24-well plates with 40,000 cells/well in regular medium. At 10–14 h postplating, the medium was replaced with regular medium containing varying amounts of concentrated virus. The next day, 1 ml regular medium was added to each well. Three days after transduction, the medium was replaced with 300 µl PBS to allow better visualization of the GFP fluorescence, and transduction efficiency was calculated based on the percentage of GFP-positive cells for the different virus dilutions. The MOIs used for COS-7 cells and primary hippocampal neurons are arbitrary MOIs based on the HEK-293–T cell transduction rate.

### Statistical Analyses

Statistical tests and posttests used are indicated in the figure legends where appropriate. *N* indicates the number of independent repeats analyzed, and *n* indicates the size of the total pool analyzed, if applicable.

### KD, Transfection, and Rescue

For KD studies in COS-7 cells, cells were plated on the day of transduction. For transduction, the culture medium was replaced by DMEM High Glucose (Invitrogen) supplemented with 2% heat-inactivated FBS, 100 U/ml penicillin, 100 µg/ml streptomycin, and 6 µg/ml polybrene (Sigma), and viruses were added at an MOI of 10. The next day, media was replaced with fresh culture medium and the cells were cultivated until assays were performed 6 d after transduction. In some cases, COS-7 KD cells were transfected using jetPRIME (Polyplus Tranfection) following the manufacturer's instruction 5 d after transduction and processed for immunofluorescence following overnight incubation. For rescue studies, KD cells were plated on coverslips on day 5 after transductions. On the same day, cells were transduced with rescue viruses using an MOI of 4 as described above. The media was replaced early the next morning, and cells were processed for immunofluorescence 24 h after the second transduction.

### Immunofluorescence

For analysis of KD COS-7 cells, cells plated on poly-L-lysine-coated coverslips were processed for immunofluorescence following standard protocols 6 d after transduction with for COS-7 cells. COS-7 cells transduced with protein expression viruses or transfected with expression constructs were processed 1 or 2 d after manipulation. Images were analyzed using Image J (National Institutes of Health, Bethesda, MD).

### 3D Superresolution Microscopy

Control and NECAP 1 KD cells (nt220) were plated with 40,000 cells/well on poly-L-lysine-coated 8-well Lab-Tek II chambered coverglasses #1.5 (cat. no. 155409). The following day, cells were fixed with 2% paraformaldehyde for 10 min at RT, processed for immunofluorescence detection of endogenous AP-2 using Alexa647-conjugated secondary antibodies, and stored in PBS until imaging. Images were recorded with a SR 200 microscope (Vutara, Inc., Salt Lake City, UT) based on the Biplane FPALM approach [55]. The system features four laser lines (405, 488, 561, and 647 nm) for excitation and activation of single fluorescent molecules. Speckle-free illumination with an even intensity distribution is realized by a specialized beam homogenizer. Images of fluorescing molecules are recorded with a 60×/1.2NA Olympus water immersion objective on an Photometrics Evolve 512 EM-CCD camera with the gain set at 50. Each acquisition consisted of 7,000 frames recorded at a speed of 40 frames/s, which encompassed a 20×20 µm field of view. The maximum powers used for the readout laser (647 nm) and activation laser (405 nm) were 4 and 0.05 kW/cm^2^, respectively.

The calibration entails experimentally calculating the point spread function (PSF) in three dimensions. This was done using 100 nm Tetraspeck beads. The analysis and rendering were done using Vutara's SRX localization and visualization software, based on an enhanced implementation of Juette et al. [55] and Mlodzianoski et al. [56]. Data were analyzed by the Vutara SRX software (version 3.16). In short, particles were identified by their brightness from the combined images taken in both planes simultaneously. If a particle was identified in multiple subsequent camera frames, data from these frames were combined for the specific identified particle. Background can be optionally removed based on the observed signal in the frames before or after the frames in which a particle was observed. Identified particles were then localized in three dimensions by fitting the raw data in a customizable region of interest (typically 16×16 pixels) centered around each particle in each plane with a 3D model function that was obtained from recorded bead datasets. The recorded fields are aligned automatically by computing the affine transformation between the pair of planes. Sample drift can be corrected by cross-correlation of the determined localized particles [56] or tracking of fiduciary markers. Fit results were stored as data lists for further analyses. The SRX software allows the 3D display of localized particles as solid shaded spheres or as an accumulation of transparent gaussian kernels. Alternatively, 2D slices through the 3D volume in any of the three main directions can be shown.

### Transferrin and EGF Internalization Assays

COS-7 cells transduced with control and NECAP 1 KD viruses were starved in DMEM High Glucose overnight. For microscopic analysis, cells were chilled on ice for 30 min and then incubated with Cy3-transferrin (200 µg/ml) in ice-cold DMEM on ice for 1 h. Cells were washed with cold PBS and incubated in prewarmed culture medium at 37°C for the times indicated. At each time point, a sample of cells was chilled on ice, surface-bound transferrin was removed by acid wash (0.2 M acetic acid, 0.5 M NaCl), followed by a PBS wash. The cells were fixed with 4% PFA and processed for immunofluorescence detection of marker proteins if indicated. For FACS analysis, cells were chilled on ice for 30 min and then incubated with Alexa633-transferrin (200 µg/ml) in ice-cold DMEM on ice for 1 h. Cells were washed with cold PBS and incubated in prewarmed culture medium at 37°C for the times indicated. At each time point, a sample of cells was chilled on ice, surface-bound transferrin was removed by acid wash (0.2 M acetic acid, 0.5 M NaCl), followed by a PBS wash. The cells were removed from the plate in 1 ml of PBS by pipetting, filtered through a cell strainer, and analyzed by flow cytometry on a FACSCalibur (Becton Dickinson). For EGF internalization assays, control and NECAP 1 KD cells were maintained in regular culture medium at 37°C. The regular medium was replaced with prewarmed DMEM High Glucose medium containing 2 ng/ml of Texas Red-labeled EGF for 2.5 min at 37°C. The cells were then chilled on ice and surface-bound EGF was removed by acid wash, followed by a PBS wash. The cells were fixed with 4% PFA, and internalized Texas Red-EGF was detected by fluorescence microscopy. For comparison, parallel sets of cells were processed to detect Cy3-transferrin (200 µg/ml) internalization under these condition.

### EM

COS-7 cell monolayers were washed in 0.1 M sodium cacodylate buffer (Electron Microscopy Sciences) and fixed in 2.5% glutaraldehyde (Electron Microscopy Sciences) in sodium cacodylate buffer overnight at 4°C. The following day the cells were washed in 0.1 M sodium cacodylate buffer and incubated in 1% osmium tetroxide (Mecalab) for 1 h at 4°C. The cells were dehydrated in a graded series of ethanol/deionized water solutions from 50%–100%. The cells were then infiltrated with a 1∶1 and 3∶1 Epon 812 (Mecalab)∶ethanol mixture, each for 30 min, followed by 100% Epon 812 for 1 h for embedding in the wells, and polymerized overnight in an oven at 60°C. The polymerized blocks were trimmed and 100 nm ultrathin sections cut with an UltraCut E ultramicrotome (Reichert Jung) and transferred onto 200-mesh copper grids (Electron Microscopy Sciences) having formvar support film. Sections were poststained for 8 min with 4% uranyl acetate (Electron Microscopy Sciences) and 5 min with lead citrate (Fisher Scientific). Samples were imaged with a FEI Tecnai 12 transmission electron microscope (FEI Company) operating at an accelerating voltage of 120 kV and equipped with a Gatan 792 Bioscan 1k61k Wide Angle Multiscan CCD Camera (Gatan, Inc.). Vesicle and pit size was measured using Image J (National Institutes of Health, Bethesda, MD).

### NMR

PHear GST fusion protein and Ex His-tag fusion protein were expressed in the *E. coli* strain BL21. To generate uniformly ^15^N-labeled protein, the cells were grown in M9 minimal media containing ^15^NH_4_Cl. Bacteria were induced at 30°C for 4 h using IPTG at a final concentration of 1 mM once OD at 600 nm reached 0.8. The GST-fusion protein was purified, cleaved with thrombin in PBS, and thrombin was removed using benzamidine-Sepharose. The protein samples were further purified by S75 gel filtration equilibrated with PBS. The Ex His-tag fusion protein was purified by Ni-charged chelating Sepharose in 8 M guanidium chloride in PBS and eluted in 2 M guanidinium chloride in PBS with 500 mM imidazol. The sample was then purified by gel filtration using a S75 column equilibrated with PBS, immediately cleaved by TEV protease, then purified by reverse phase HPLC using a C18 column. The purified protein was lyophilized to afford a white powder. The NMR samples contain freshly prepared buffer with 25 mM sodium phosphate pH 7.2, 75 mM NaCl, 0.5 mM EDTA, and 3 mM DTT. All NMR experiments were performed at 30°C using a Bruker DRX 600-MHz spectrometer. Spectra were processed by NMRPipe [57] and analyzed by NMRview [58]. NMR titrations were carried out by acquiring ^1^H-^15^N heteronuclear single quantum correlation (HSQC) spectra on 250 µL of ^15^N-labeled protein at a concentration of 0.1–0.2 mM. Subsequent spectra were taken after the addition of an unlabeled ligand. Analysis of peptide binding to the PHear domain was carried out by comparison of chemical shifts for backbone amide signals in ^15^N–^1^H HSQC spectra. Weighted average shifts ((Δδ^15^N)_2_+(Δδ^1^H)_2_)0.5 were used to identify binding site residues. The NMR assignments for the NECAP 1 PHear domain were previously determined [16]. Peptides for AP-180 (Ac-VDIFGDAFAAS) and the β2-linker (Ac-SQGDLLGDLLNLDLPPVN) were purified by reverse phase C18 HPLC, lyophilized, and dissolved in NMR buffer to make peptide concentrations of 4.1 mg/mL and 5.1 mg/mL, respectively. ^15^N-labeled PHear at a concentration of 0.15 mM was titrated with unlabeled peptides at relative concentrations of 2∶1, 1∶1, 1∶2, 1∶4, 1∶8, and 1∶16. To observe that the PHear domain competes with clathrin for access to the β2-linker, ^15^N-labeled PHear together with unlabeled β2-linker peptide were titrated with unlabeled clathrin terminal domain (aa 1–579). The relative concentrations of PHear, β2-linker, and clathrin terminal domain were 1∶0∶0, 1∶4∶0, 2∶8∶1, 1∶4∶1, and 1∶4∶2, respectively.

### CCV Purification and Isolation of Coat Proteins

CCVs were purified from adult rat brain using buffer A (100 mM MES, pH 6.5, containing 1 mM EGTA, and 5 mM MgCl_2_) as described previously [59]. For the extraction of coat proteins, CCVs were centrifuged at 200,000× *g* for 15 min, the pellet was resuspended in 0.5 M Tris pH 7.0, 2 mM EDTA, and incubated for 30 min on ice. The samples were centrifuged at 200,000× *g* and the supernatant fraction was resolved by SDS-PAGE, transferred to nitrocellulose and processed for Western blotting or overlay assays.

### GST Affinity Selection Assays, Co-Immunoprecipitation, and Overlays

For affinity selection assays, soluble cell and rat brain extracts for affinity selection assays were prepared in 10 mM HEPES, pH 7.4, 1% Triton X-100, 50 mM NaCl, 0.83 mM benzamidine, 0.23 mM phenylmethylsulphonyl fluoride, 0.5 µg/ml aprotinin, and 0.5 µg/ml leupeptin and incubated for 1 h at 4°C with GST fusion proteins precoupled to glutathione-Sepharose. For AP-2 co-immunoprecipitation assays from 293-T cell lysates transfected with Flag-tagged NECAP 1 variants, the NaCl concentration in the buffer was reduced to 33 mM and lysates were incubated with protein G-agarose alone (mock) or with protein G-agarose and 10 µg Flag-antibody (M2) for 1 h at 4°C. Co-immunoprecipitation studies of endogenous AP-2 from brain cytosol and solubilized membrane fraction were performed as described previously [19]. Proteins were resolved by SDS-PAGE and analyzed by Western blotting. Overlays (far-Western blots) were performed as described previously [60].

### Competition Assay

CCVs were purified from rabbit brain (Pel-Freez, 250 g) and coat proteins were prepared for gel filtration as previously described [61]. Coat proteins were separated on a Hiprep 26/60 Sephacryl S-300 HR size exclusion column and eluted at 0.5 ml/min. Fractions of 1 ml were collected, AP-2-containing fractions identified by Western blot, and stored at −80°C until use. For competition assays, one 1 ml fraction was precipitated with ammonium sulfate, spun for 30 min at 10,000× *g*, and the resulting pellet dissolved in binding buffer (10 mM HEPES, pH 7.4, 1% Triton X-100, 50 mM NaCl, 0.83 mM benzamidine, 0.23 mM phenylmethylsulphonyl fluoride, 0.5 µg/ml aprotinin, and 0.5 µg/ml leupeptin). Equal aliquots of the purified AP-2 solution were incubated for 1 h at 4°C with 40 µg of purified GST-clathrin terminal domain or GST alone precoupled to glutathione beads. Unbound proteins were removed by washing three times with 1 ml of binding buffer. Equal fractions of GST-clathrin terminal domain were incubated 1 h at 4°C with either 1 ml of binding buffer alone or with 1 ml of binding buffer supplemented with varying amounts of purified PHear–Ex. Unbound proteins were removed as before and the samples were resolved by SDS-PAGE, and AP-2 binding to the clathrin terminal domain was analyzed by Western blot.

## Supporting Information

Figure S1 **Endogenous NECAP 1 expression in cultured cell lines.** Western blot analysis of the expression levels of endogenous NECAP 1 in cultured cell lines as indicated. For each cell line, 150 µg of total cell lysate were analyzed.
(PDF)

Figure S2 **Vesicle formation sites increase in size upon NECAP 1 KD.** 3D superresolution analysis of AP-2-labeled vesicle formation sites in control and NECAP 1 KD cells. Each vesicle formation site is shown in x/y, x/z, and y/z orientation with signals rendered to 5 nm particle size. Each colored box contains five structures analyzed for four control (purple background) and NECAP 1 KD cells (green background). The scale bar represents 100 nm.
(PDF)

Figure S3 **Vesicle formation sites at both the cell surface contacting the cover slip and the cell surface facing the culture medium increase in size upon NECAP 1 KD.** (A) Serial z-sections of confocal images of AP-2-labeled vesicle formation sites in control (shRNAmiR-control) and NECAP 1 KD (nt220) cells. The boxed areas are shown in higher magnification in the bottom two rows. (B) 3D superresolution analysis of AP-2-labeled vesicle formation sites in NECAP 1 KD cells at the bottom surface (cell surface contacting the coverslip) and top surface (cell surface facing the culture medium) of cells. Each vesicle formation site is shown in x/y, x/z, and y/z orientation with signals rendered to 5 nm particle size.
(PDF)

Figure S4 **Synaptic vesicles increase in size upon NECAP 1 KD.** (A) Equal protein amounts of lysates from primary hippocampal neurons transduced with control virus (shRNAmiR-control) or viruses to KD NECAP 1 (NECAP 1 nt298 and NECAP 1 nt685) were tested by Western blot for the indicated proteins. (B) Representative electron micrographs of synapses from primary hippocampal neurons transduced as indicated. The bar represents 200 nm. (C and D) Analysis of the average diameter of synaptic vesicles in synapses of control and NECAP 1 KD neurons. Repeated measures one-way ANOVA followed by Bonferroni's Multiple Comparison Test revealed significant differences, *p*<0.002, *N* = 3. (C) The bar graph shows the distribution of the average vesicle diameter per synapse within the whole population of synapses analyzed in each condition. (D) Bar graph representing the mean synaptic vesicle diameter of all synapses analyzed. Repeated measures one-way ANOVA followed by Bonferroni's Multiple Comparison Test revealed significant differences as indicated, **p = 0.0018, *N* = 3. (E) Bar graph representing the average number of synaptic vesicles per synapse. Repeated measures one-way ANOVA followed by Bonferroni's Multiple Comparison Test revealed significant differences as indicated, *p = 0.023, *N* = 3.
(PDF)

Movie S1 **3D superresolution representation of AP-2-labeled vesicle formation sites within a 4 µm^2^ area of a control cell with signals rendered to 50 nm particle size.**
(MOV)

Movie S2 **3D superresolution representation of control AP-2-labeled vesicle formation sites selected from Movie S1 with signals rendered to 50 nm particle size.**
(MOV)

Movie S3 **3D superresolution representation of AP-2-labeled vesicle formation sites within a 4 µm^2^ area of a NECAP 1 KD cell with signals rendered to 50 nm particle size.**
(MOV)

Movie S4 **3D superresolution representation of NECAP 1 KD AP-2-labeled vesicle formation sites selected from Movie S3 with signals rendered to 50 nm particle size.**
(MOV)

## Abbreviations

CCP: clathrin-coated pit
CCV: clathrin-coated vesicle
EGF: epidermal growth factor
GST: glutathione-S-transferase
KD: knockdown
PH: pleckstrin homology
PHear: PH domain with ear-like function
