# Supplementary figures and images for: NECAP 1 Regulates AP-2 Interactions to Control Vesicle Size, Number, and Cargo During Clathrin-Mediated Endocytosis
Source: PLoS Biol. 2013 Oct 1;11(10):e1001670. doi: 10.1371/journal.pbio.1001670 (PMC3794858; doi:10.1371/journal.pbio.1001670)

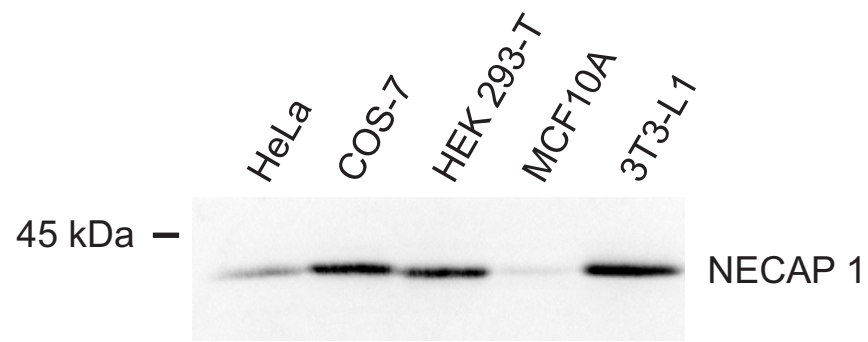

Supplement: Figure S1 — Endogenous NECAP 1 expression in cultured cell lines. Western blot analysis of the expression levels of endogenous NECAP 1 in cultured cell lines as indicated. For each cell line, 150 µg of total cell lysate were analyzed. (PDF) [file pbio.1001670.s001.pdf]

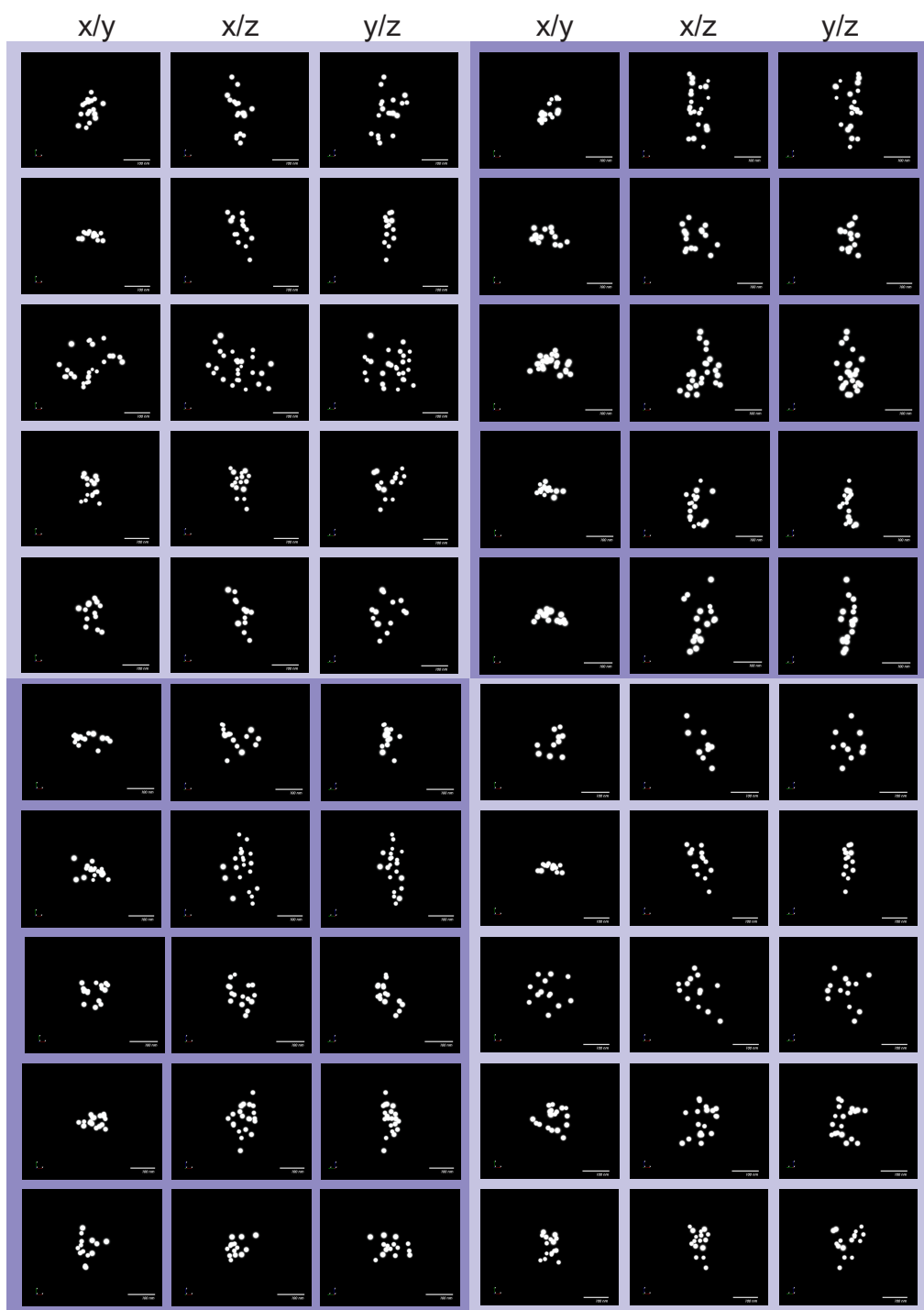

shRNA*miR*-control

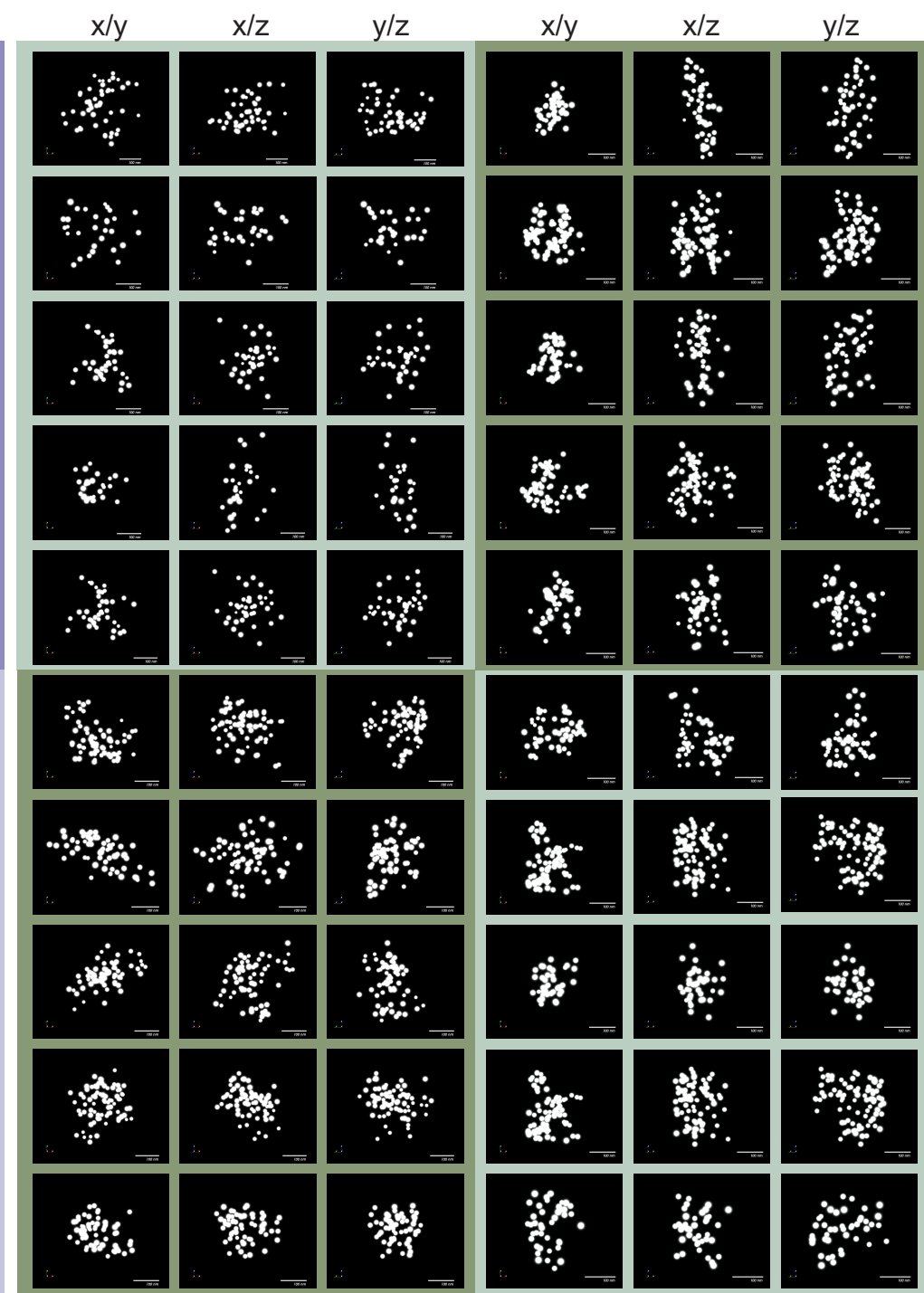

NECAP 1 nt220

Supplement: Figure S2 — Vesicle formation sites increase in size upon NECAP 1 KD. 3D superresolution analysis of AP-2-labeled vesicle formation sites in control and NECAP 1 KD cells. Each vesicle formation site is shown in x/y, x/z, and y/z orientation with signals rendered to 5 nm particle size. Each colored box contains five structures analyzed for four control (purple background) and NECAP 1 KD cells (green background). The scale bar represents 100 nm. (PDF) [file pbio.1001670.s002.pdf]

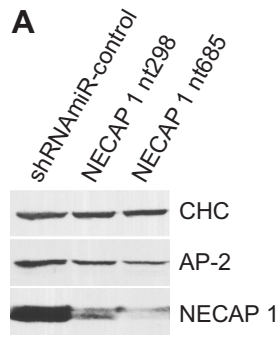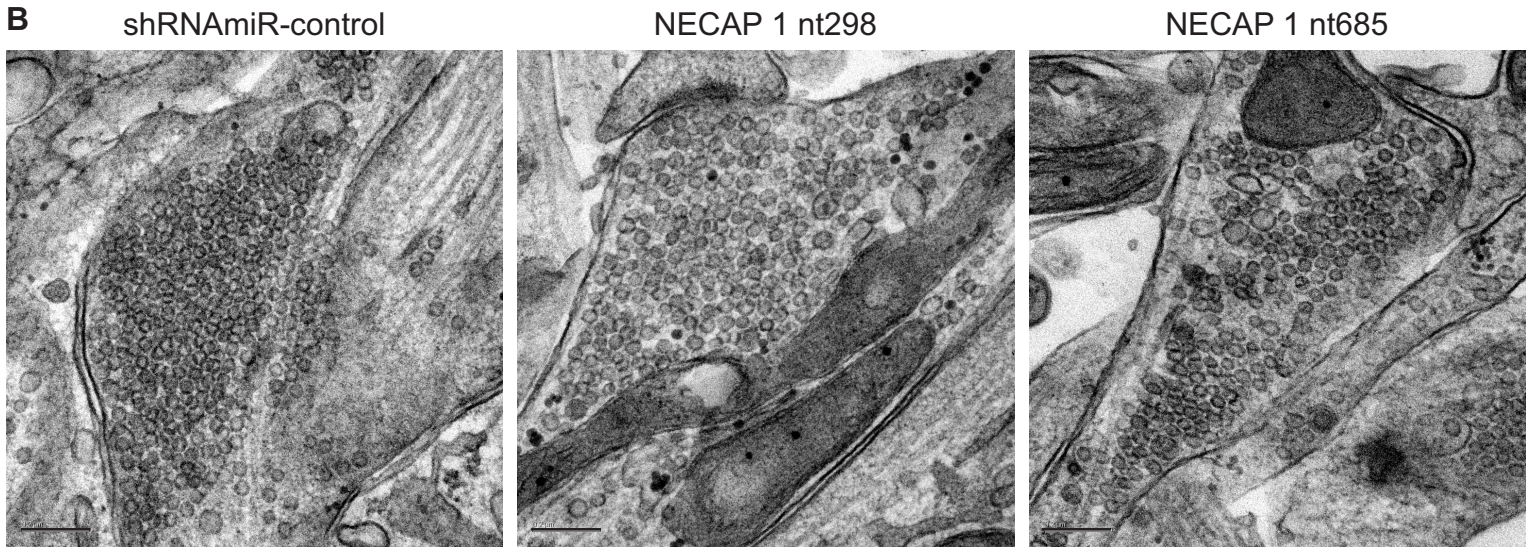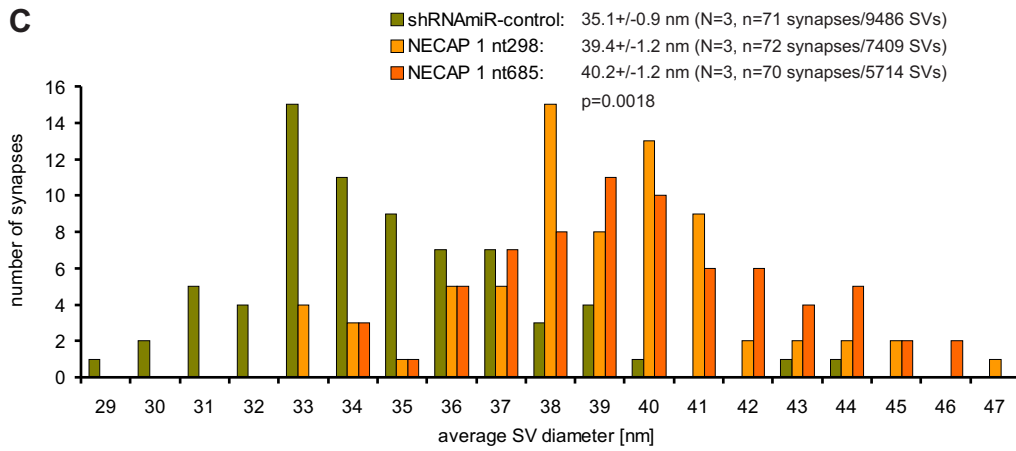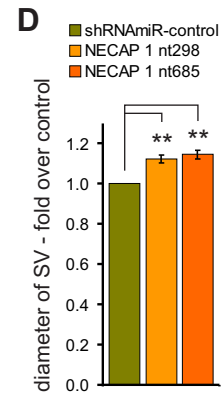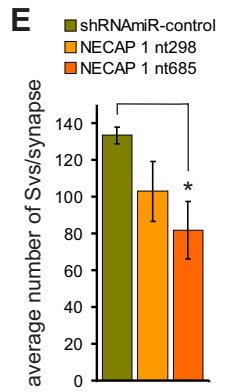

Supplement: Figure S4 — Synaptic vesicles increase in size upon NECAP 1 KD. (A) Equal protein amounts of lysates from primary hippocampal neurons transduced with control virus (shRNAmiR-control) or viruses to KD NECAP 1 (NECAP 1 nt298 and NECAP 1 nt685) were tested by Western blot for the indicated proteins. (B) Representative electron micrographs of synapses from primary hippocampal neurons transduced as indicated. The bar represents 200 nm. (C and D) Analysis of the average diameter of synaptic vesicles in synapses of control and NECAP 1 KD neurons. Repeated measures one-way ANOVA followed by Bonferroni's Multiple Comparison Test revealed significant differences, p<0.002, N = 3. (C) The bar graph shows the distribution of the average vesicle diameter per synapse within the whole population of synapses analyzed in each condition. (D) Bar graph representing the mean synaptic vesicle diameter of all synapses analyzed. Repeated measures one-way ANOVA followed by Bonferroni's Multiple Comparison Test revealed significant differences as indicated, **p = 0.0018, N = 3. (E) Bar graph representing the average number of synaptic vesicles per synapse. Repeated measures one-way ANOVA followed by Bonferroni's Multiple Comparison Test revealed significant differences as indicated, *p = 0.023, N = 3. (PDF) [file pbio.1001670.s004.pdf]
