# Supplementary material for: NECAP 1 Regulates AP-2 Interactions to Control Vesicle Size, Number, and Cargo During Clathrin-Mediated Endocytosis
Source: PLoS Biol. 2013 Oct 1;11(10):e1001670. doi: 10.1371/journal.pbio.1001670 (PMC3794858; doi:10.1371/journal.pbio.1001670)
Supplement: Figure S3 — Vesicle formation sites at both the cell surface contacting the cover slip and the cell surface facing the culture medium increase in size upon NECAP 1 KD. (A) Serial z-sections of confocal images of AP-2-labeled vesicle formation sites in control (shRNAmiR-control) and NECAP 1 KD (nt220) cells. The boxed areas are shown in higher magnification in the bottom two rows. (B) 3D superresolution analysis of AP-2-labeled vesicle formation sites in NECAP 1 KD cells at the bottom surface (cell surface contacting the coverslip) and top surface (cell surface facing the culture medium) of cells. Each vesicle formation site is shown in x/y, x/z, and y/z orientation with signals rendered to 5 nm particle size. (PDF) [file pbio.1001670.s003.pdf]

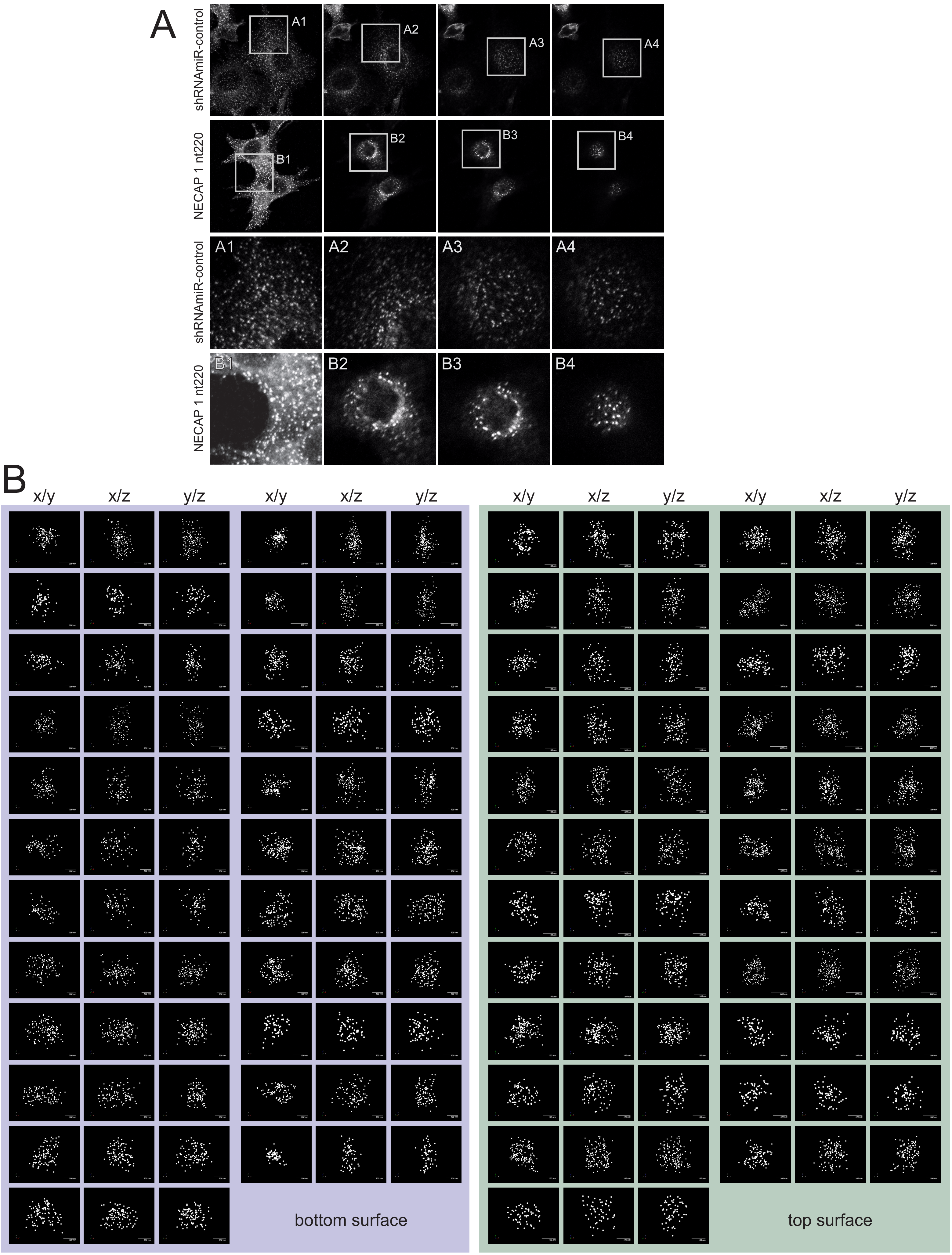
A
shRNAmiR-control
NECAP 1 nt220
shRNAmiR-control
NECAP 1 nt220
A1
A2
A3
A4
B1
B2
B3
B4
B
x/y
x/z
y/z
x/y
x/z
y/z
x/y
x/z
y/z
x/y
x/z
y/z
bottom surface
top surface
